# Supplementary material for: Thyroid Hormone Receptor Beta in the Ventromedial Hypothalamus Is Essential for the Physiological Regulation of Food Intake and Body Weight
Source: Cell Rep. 2017 Jun 13;19(11):2202–9. doi: 10.1016/j.celrep.2017.05.066 (PMC5478879; doi:10.1016/j.celrep.2017.05.066)
Supplement: Document S1. Supplemental Experimental Procedures, Figures S1–S4, and Table S3 [file mmc1.pdf]

Cell Reports, Volume *19*

Supplemental Information

Thyroid Hormone Receptor Beta in the Ventromedial Hypothalamus Is Essential for the Physiological Regulation of Food Intake and Body Weight

Saira Hameed, Michael Patterson, Waljit S. Dhillo, Sofia A. Rahman, Yue Ma, Christopher Holton, Apostolos Gogakos, Giles S.H. Yeo, Brian Y.H. Lam, Joseph Polex-Wolf, Wiebke Fenske, Jimmy Bell, Jelena Anastasovska, Jacques Samarut, Stephen R. Bloom, J.H. Duncan Bassett, Graham R. Williams, and James V. Gardiner

A

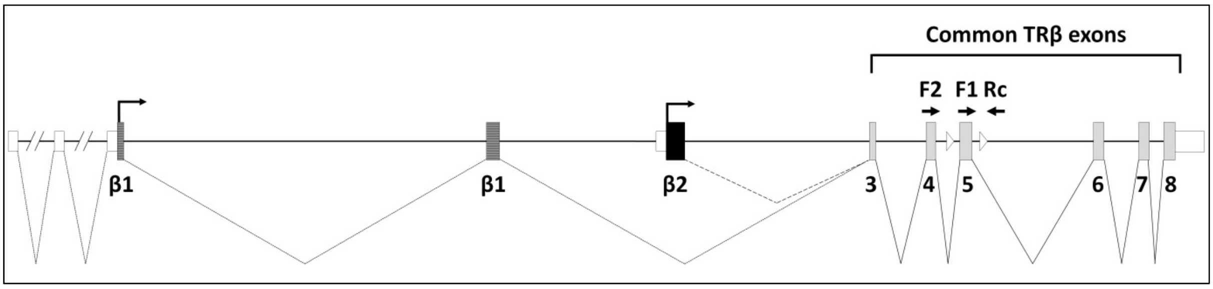

B

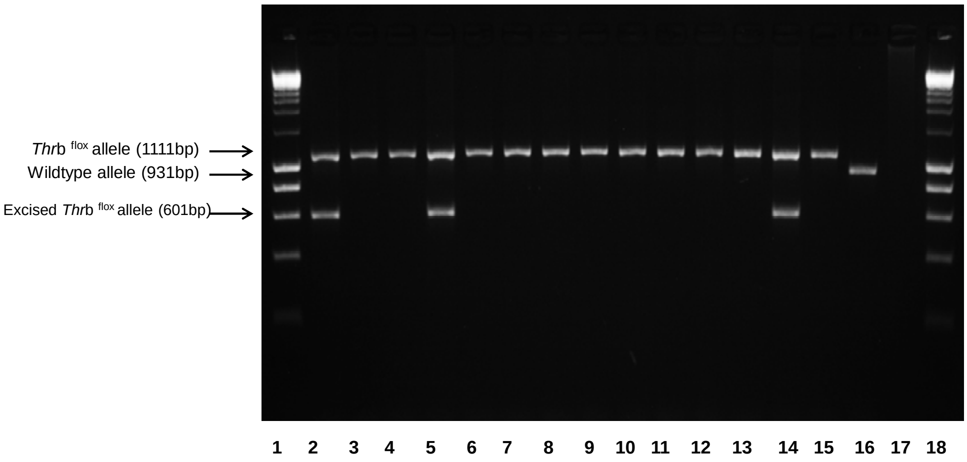

Supplemental Data Items

Figure S1 Schematic representation of the *Thrb*^{flox} allele and verification of excision of the *Thrb*^{flox} allele related to figure 1

A) Genomic structure of *Thrb* (NC_000080.6 (17660960-18038088)) showing the locations of the thyroid hormone receptor beta 1 (*TRβ1:* NM_001113417.1) and beta 2 (*TRβ2:* NM_009380.3) isoforms. White boxes represent untranslated exons, shaded boxes indicate unique 5' *Thrβ1* exons, the black box shows the unique 5' *Thrβ2* exon, and the light grey boxes show the 6 exons common to both isoforms. The positions of the two LoxP sites flanking exon 5 are indicated by the white triangles. The wild type and *Thrb*^{flox} alleles were amplified using the forward (F1: 5'-CAGCCACTGGAAGCAGAAG-3') and reverse primers (Rc: 5'-AACGTCACTGTTGTGGTGTACAGG-3'). PCR amplification of the wild type allele resulted in a 931bp product whereas the product of the *Thrb*^{flox} allele was 1111bp in size. The excised *Thrb*^{flox} allele was amplified using the forward primer (F2: 5'-CATCTATGTTGGCATGGCAACAGACT-3') and reverse primer (Rc) the resulting product being 601bp in size.

B) Agarose gel visualized under UV illumination of PCR on DNA to demonstrate excision of the *Thrb*^{flox} allele in *Thrb*$^{flox/flox}$ mice, restricted to the hypothalamus, following intra-VMH injection of rAAV-Cre. Arrows donate position of *Thrb*^{flox} allele (1111bp), wildtype allele (931bp) and the excised *Thrb*^{flox} allele (601bp). Lane 1: HyperLadder I™ (DNA molecular weight marker). Lanes 2 and 5: hypothalamic DNA from two *Thrb*$^{flox/flox}$ mice injected with rAAV-Cre into the VMH, denoted as VMH-TRβ^{-} mouse 1 and VMH-TRβ^{-} mouse 2 respectively, each showing a band at 1111bp representing the *Thrb*^{flox} allele and a band at 601bp representing the excised *Thrb*$^{\Delta VMH}$ allele. Lanes 3 and 6: PCR performed on DNA extracted from the cerebellum (lane 3, VMH-TRβ^{-} mouse 1; lane 6, VMH-TRβ^{-} mouse 2). Lanes 4 and 7: PCR performed on DNA extracted from and brainstem (lane 4, VMH-TRβ^{-} mouse 1; lane 7, VMH-TRβ^{-} mouse 2). In these lanes only the band at 1111bp representing the *Thrb*^{flox} allele is present. The absence of a band at 601bp in these lanes demonstrates that the *Thrb*^{flox} allele has not been excised in these extra-hypothalamic brain tissues. Lanes 8 and 11: hypothalamic DNA from two *Thrb*$^{flox\ /flox}$ mice injected with rAAV-GFP into the VMH, denoted as VMH-GFP-mouse 1 and VMH-GFP-mouse 2 respectively, each showing a band at 1111bp representing the *Thrb*^{flox} allele. The absence of a band at 601bp in these lanes demonstrates that the *Thrb*^{flox} allele has not been excised in the hypothalami of the rAAV-GFP injected mice. Lanes 9 and 12: PCR performed on DNA extracted from the cerebellum (lane 9, VMH-GFP-1-mouse 1; lane 12, VMH-GFP-mouse 2). Lanes 10 and 13: PCR performed on DNA extracted from the brainstem (lane 10, VMH-GFP-mouse 1; lane 13, VMH-GFP-mouse 2). Lane 14: PCR of DNA extracted from the hypothalamus of a *Thrb*$^{flox/flox}$ mouse injected with rAAV-Cre. Lane 15: PCR of DNA extracted from the hypothalamus of an un-injected *Thrb*$^{flox/flox}$ mouse. Lane 16: PCR of DNA extracted from the hypothalamus of a wildtype mouse. Lane 17: negative control (autoclaved glass distilled water). Lane 18: HyperLadder I™ (DNA molecular weight marker).

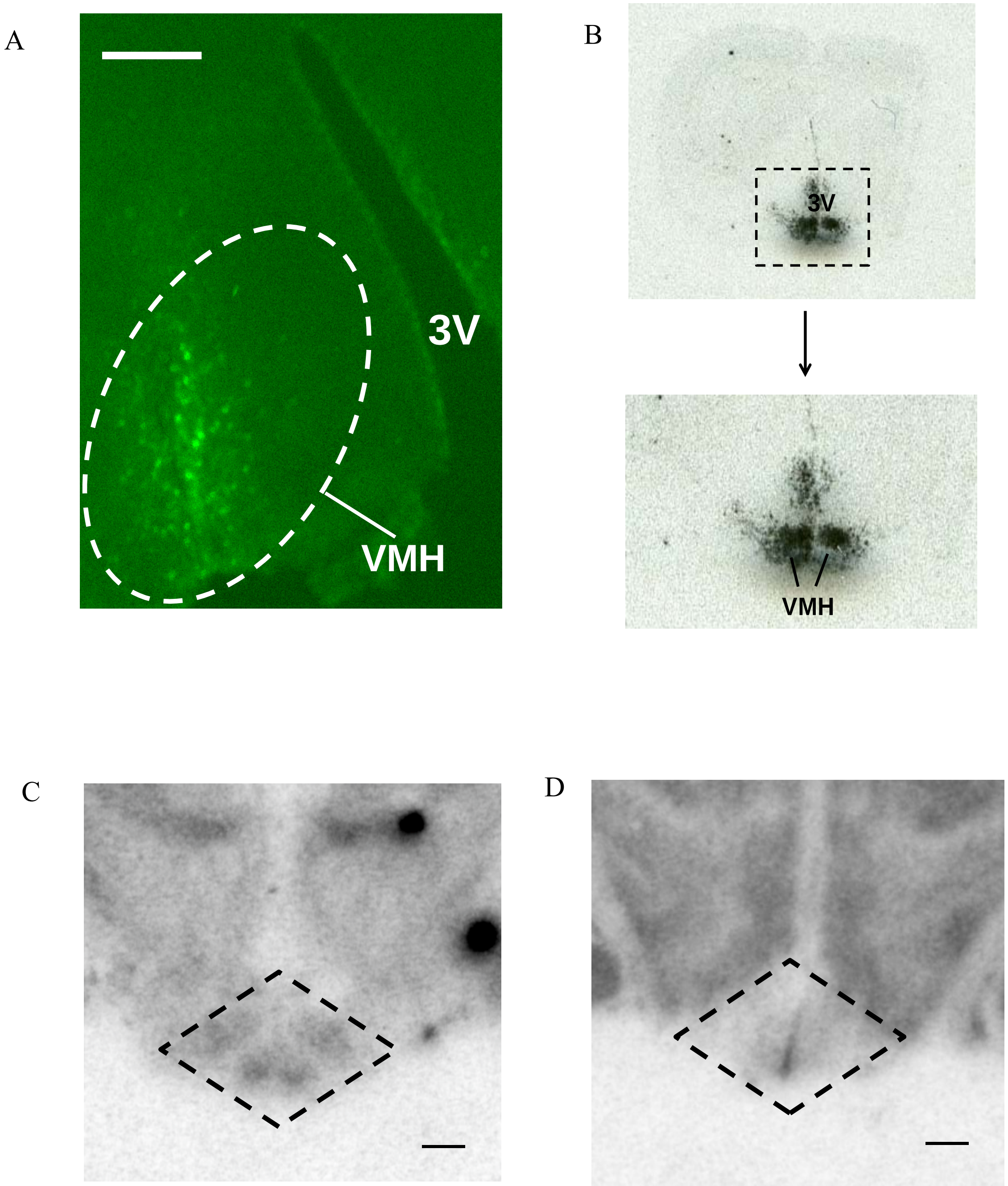

Figure S2 Localization studies, verification of rAAV-Cre transgene expression and excision of the TRβ^{flox} allele within the ventromedial hypothalamus related to figure 1

(A) GFP fluorescence within the VMH which sits adjacent to the third ventricle (3V) (scale bar represents 6μm). **(B)** Representative *in situ* hybridization image of a VMH-TRβ$^-$ mouse brain radio-labelled with woodchuck hepatitis post-regulatory element (WPRE) antisense riboprobe which localizes transgene expression to the VMH. The WPRE sequence is part of the expression cassette of rAAV vectors but is not endogenously expressed by mammalian cells. Its detection therefore confirms successful rAAV neuronal infection and transgene expression. Scale bar is 0.2mm **(C)** Representative *in situ* hybridization image of a VMH-GFP mouse brain radio-labelled with TRβ antisense riboprobe showing expression of TRβ within the VMH (hashed area). This is in comparison to the *in situ* hybridization image **(D)** showing a VMH-TRβ$^-$ mouse brain radio-labelled with *Thrb* antisense riboprobe. The lack of riboprobe binding in the VMH-TRβ$^-$ mouse brain (D) (area marked by hashed lines) in comparison to the control brain (C) suggests reduced expression of TRβ in the VMH of VMH-TRβ$^-$ mice. Scale bar 25μm.

A

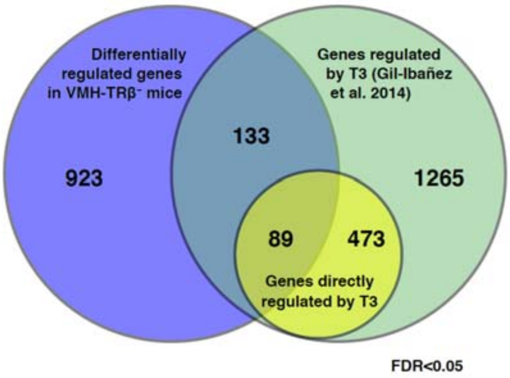

B

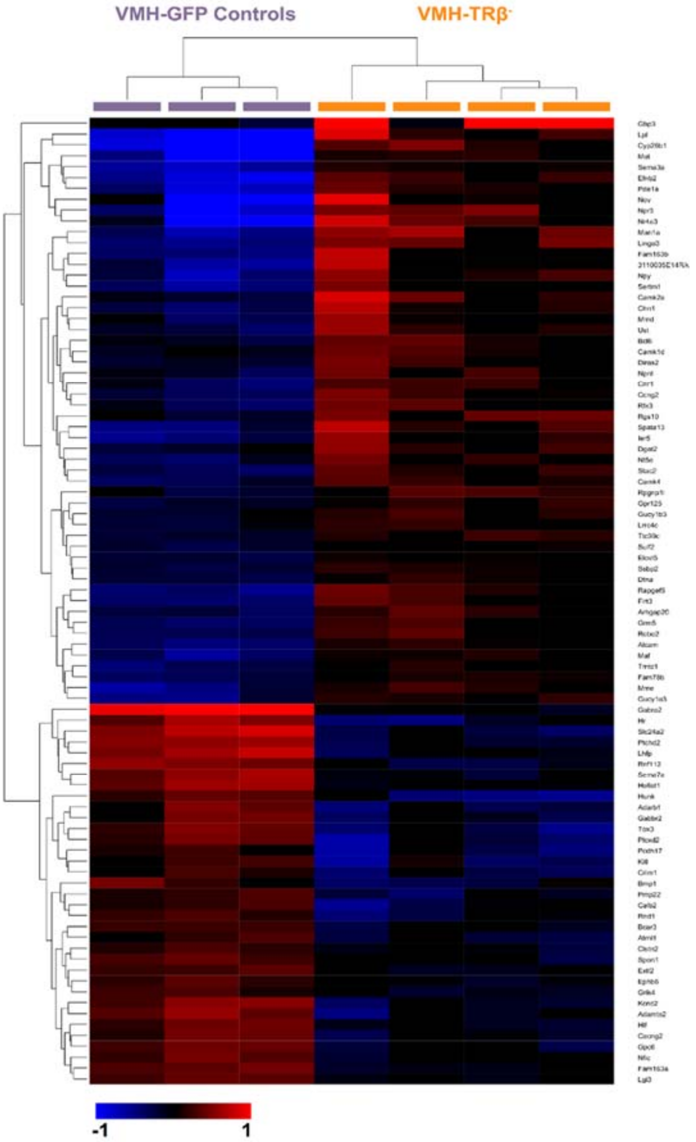

Figure S3: Venn diagram of comparison of differentially regulated gens in VMH-TRβ- mice and those reported to be T3 sensitive in cerebrocortical cells and heat map of significantly altered genes directly regulated by T3 related to figure 2

RNA was extracted from hypothalami from VMH-GFP (n=3) and VMH-TRβ- (n=4) mice and subject to RNA seq analysis.

A. Significant differential expression genes were compared to those reported by Gil-Ibañez al 2017.

B. Significant differential expression genes were compared to those reported by Gil-Ibañez al 2017 and shown are the 93 genes reported to be directly regulated by T3 that were significantly altered in our samples.

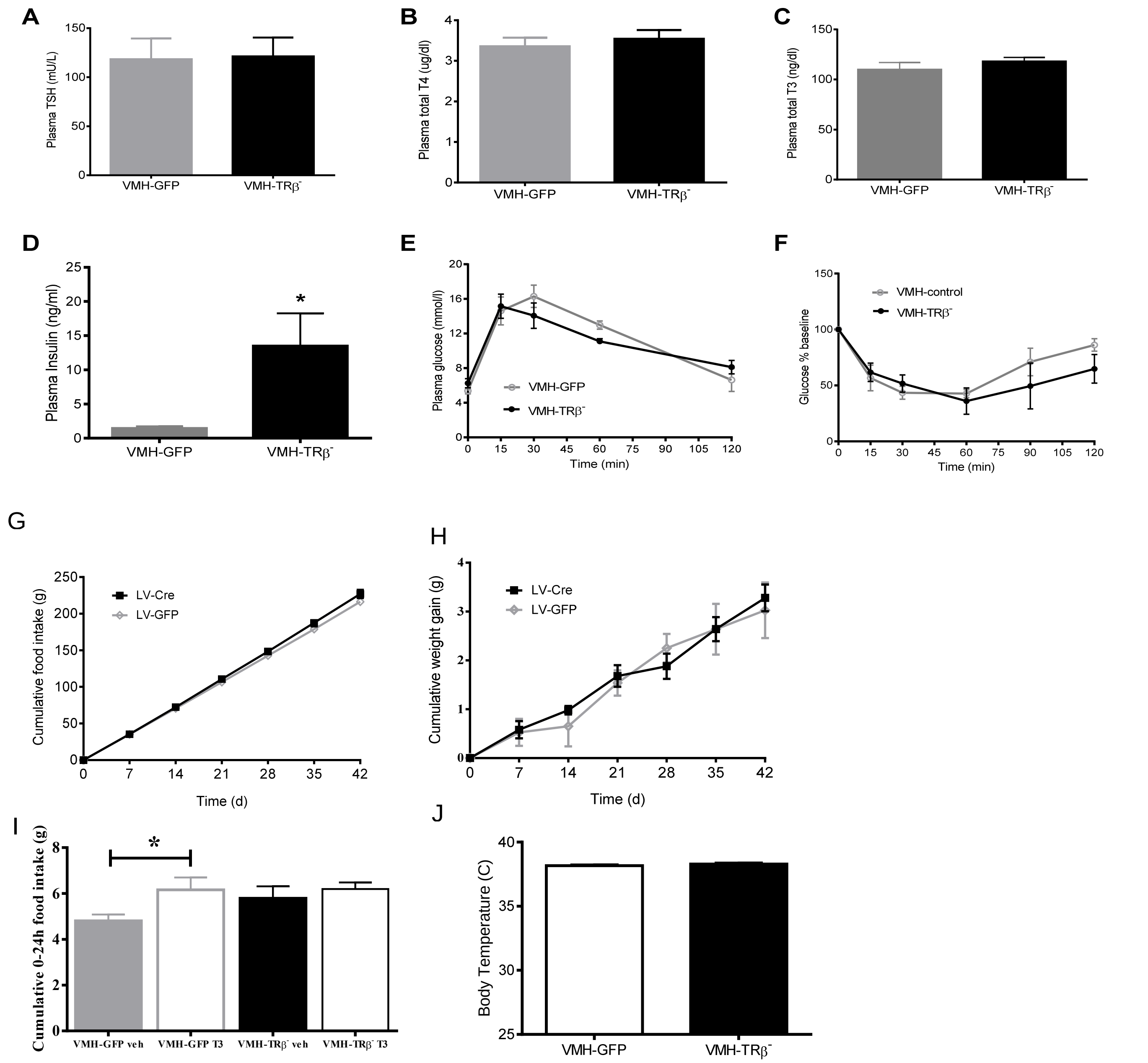

Figure S4: Systemic thyroid function and glucose homeostasis body temperature and response to exogenous T3 injection of VMH-TRβ^{-} and VMH-GFP and effect of lateral ventricle injection of rAAV-Cre (LV-Cre) or rAAV-GFP (LV-GFP) into the lateral ventricles (LV) of *Thrb*$^{flox/flox}$ mice mice related to figure 2

(A) Plasma mTSH.

(B) Plasma total T4.

(C) Plasma total T3.

(D) Fasting plasma insulin.

(E) Glucose tolerance test performed before development of obesity in mice.

(F) Insulin tolerance test performed before development of obesity in mice.

(G) Weight change in LV-Cre and LV-GFP mice

(H) Cumulative food intake in LV-Cre and LV-GFP mice

(I) Twenty-four hour food intake in VMH-GFP or VMH-TRβ^{-} mice in response to exogenous T3 or vehicle.

(J) Body temperature in VMH-TRβ^{-} and VMH-GFP mice.

Results (A-C) are mean ± s.e.m. (n=7 per group). Data were analyzed by Mann-Whitney U test.

Results D are mean ± s.e.m. (n=10 per group). Results E-F are mean ± s.e.m. (n=9 per group).

Results G and H are mean ± s.e.m. (n=8-10 per group). Data were analyzed using generalized estimating equations with exchangeable correlation matrix and robust standard errors

Results I are mean ± s.e.m (n=5-7 per group) Results J are mean ± s.e.m (n=4 per group) Data were analyzed by t-test.

.

	Dark phase		Light phase	
	**VMH-GFP**	**VMH-TRβ$^-$**	**VMH-GFP**	**VMH-TRβ$^-$**
**Feeding**	19 (16-23)	21 (9-25)	14 (13-17)	9 (9-14)
**Drinking**	2 (0-3)	0 (0-0)	0 (0-1)	0 (0-0)
**Grooming**	24 (22-26)	17 (11-24)	15(10-19)	13 (4-16)
**Burrowing**	1 (0-2)	1 (0-3)	3 (0-4)	6 (1-8)
**Rearing**	2 (0-3)	0 (0-1)	0 (0-0)	0 (0-0)
**Locomotion**	21 (17-22)	**11 (7-13)****	9 (7-10)	6 (3-8)
**Sleep**	24 (19-24)	36 (32-41)	60 (42-65)	60 (48-68)
**Head down/still**	17 (11-21)	21 (13-32)	10 (3-15)	15 (13-18)

Table S3 Effect of TRβ inactivation in the VMH on mouse behavior related to figure 4. At least twenty eight days after rAAV injection, behavioral patterns were monitored continuously for sixty minutes at 08.30h, 12.30h, 16.30h, 19.30h, 00.00h and 04.00h by observers blinded to the experimental treatment. At every time point, each animal was observed for three five second periods every five minutes and the behavior noted. There was a significant reduction in nocturnal locomotor activity in VMH-TRβ$^-$ compared with the control group. There was no difference in abnormal behaviors (defined by a significant increase in head down, burrowing or rearing) between VMH-TRβ$^-$ and control mice. Results are median (interquartile range) (n=7-10 per group); ** P<0.01 versus control data were analyzed by Kruskal-Wallis one way analysis of variance.

Supplemental Experimental Procedures

Stereotaxic surgery
Stereotaxic surgery was performed on eight week old male *Thrb*$^{flox/flox}$ mice (Gardiner et al., 2005). The VMH injection coordinates were 1.3mm posterior, 0.4mm lateral and 6mm ventral. The LV coordinates were 0.5mm posterior, 1.1mm lateral and 2.4mm ventral. Each mouse received a 0.5 µl bilateral injection of either rAAV-Cre 7.63×10^{13} gp/ml or rAAV-GFP, 8.57×10^{13} gp/ml. Mice were individually housed at 21-23°C with a 12-h light/dark cycle with *ad libitum* access to food (RM1 diet; DS, Witham, UK) and water unless otherwise specified.

RNA seq analysis
RNA-Seq analysis was performed using hypothalamic RNA from VMH-GFP (n=3) and VMH-TRβ- (n=4) mice using Next Generation Sequencing (NGS) technologies (Imperial BRC Genomics Laboratory, Imperial College London). TruSeq Stranded mRNA libraries were multiplexed and sequenced with the average of 40 million DNA fragments per sample (100 bp paired-end reads). Quality control was performed using FastQC software (version 0.11.2). Sequencing reads were aligned to GRCm38 reference mouse genome by Tophat (version 2.0.10) using the set of known genes provided by Ensembl database (release 75) with the average alignment rate of 85%. The raw number of read pairs mapped to each Ensemble gene was calculated with HTSeq (version 0.6.0) in 'union' mode. Reads (or read pairs) that overlap more than one gene or mapped to multiple locations were discarded. Differential expression analysis was performed using EdgeR and an FDR cutoff of 0.05 was used to generate the lists of DE genes. The lists of T3 responsive genes and direct T3 responsive genes were obtained from Gil-Ibañez et al. 2017 and overlapped with DE expressed genes from the present study. Ingenuity Pathway Analysis was performed using the resultant sets of DE genes. A heatmap comparing gene expression in 89 direct T3 responsive genes in the present study was generated using GeneSpring.

Quantitation and distribution of white adipose tissue by MRI
VMH-TRβ$^-$ and VMH-GFP mice (n=3 per group) were scanned using a 4.7 Tesla Varian INOVA imaging system. SliceOmatic software (version 4.2) was used to separate and quantify tissue volumes (Mystkowski et al., 2000). Quantitation of fat depots was normalized to total body fat and total body fat was normalized to body weight.

Glucose and insulin tolerance tests
Glucose and insulin tolerance tests were carried out as previously described (Bewick et al., 2009). Plasma glucose was measured using the Acensia Contour blood glucose monitoring system (Bayer HealthCare, Newbury, U.K.).

Peripheral (subcutaneous) administration of T3 (75nmol/kg) and food intake
In a randomized crossover, VMH-TRβ$^-$ mice and VMH-GFP mice control mice (n=5-7 per group) received either subcutaneous T3 (75nmol/kg) or vehicle as previously described (Kong et al., 2004) and food intake measured.

GFP visualization
Animals were terminally anaesthetized and the brains dissected, incubated and sliced as previously described (Gardiner et al., 2005). Fluorescence was detected by a Zeiss deconvoluting microscope (Axiovert S100 TV, Carl Zeiss, Jena, Germany) using a FITC filter. Images were acquired using a MetaMorph imaging system (Universal Imaging, West Chester, USA) as previously described (Gardiner et al., 2005).

Pair-feeding of VMH-TRβ$^-$ mice to the food intake of VMH-GFP mice
Twenty eight days after rAAV injection, VMH-TRβ$^-$ mice were pair-fed to the mean daily food intake of a weight matched VMH-GFP litter mate (n=9 per group). After 5 weeks of pair-feeding, *ad libitum* feeding was re-instated for a further 4 weeks.

Plasma assays
Total T4, T3 and TSH were measured by radioimmunoassay (RIA) (Pohlenz et al., 1999). Fasting leptin and fasting insulin were measured by enzyme linked immunosorbent assay (Crystal Chem, IL).

Quantitation of *Ucp1* mRNA expression in BAT by northern blot analysis
RNA was extracted from inter-scapular BAT of VMH-TRβ$^-$ and VMH-GFP mice (n=7-11 per group) and *Ucp1* mRNA expression determined by northern blot analysis as previously described (Smith et al., 2008).

Measurement of energy expenditure
The study commenced 21 days after rAAV injection. One week and 6 weeks into the study, metabolic parameters were measured for 24h (12h light phase, 12h dark phase) by indirect calorimetry using an open-circuit Oxymax system of the Comprehensive Lab Animal Monitoring System from Columbus Instruments (Columbus, OH, USA). Animals (n=5-6 per group) were maintained at 21-23°C with a 12-h light/dark cycle with *ad libitum* access to food (RM1 diet; DS, Witham, UK) and water. To measure oxygen consumption and carbon dioxide production exhaust air from each tight chamber was sampled for 1min at 30min intervals. Oxygen consumption and carbon dioxide production were normalized to surface area (Tschop et al., 2011) (body weight to the power of 0.75). The ambulatory activity of each animal was assessed simultaneously using the optical beam technique as previously described (Gardiner et al., 2010).

Behavioral Analysis

At least 28 days after rAAV injection, behavioral patterns of VMH-TRβ^{-} and VMH-GFP mice (n=7-10 per group) were monitored continuously for sixty minutes at 08.30h, 12.30h, 16.30h, 19.30h, 00.00h and 04.00h, by observers blinded to the experimental treatment. At every time point, each animal was observed for three, five second periods every five minutes and the behavior noted. Behavior was classified into eight categories: feeding, drinking, grooming, burrowing, rearing, locomotion, sleep, head down/still as previously described (Fray et al., 1980; Abbott et al., 2001). Abnormal behavior was defined by a significant increase in head down, burrowing or rearing as previously described (Abbott et al., 2001).

Supplemental References

Abbott, C.R., Rossi, M., Wren, A.M., Murphy, K.G., Kennedy, A.R., Stanley, S.A., Zollner, A.N., Morgan, D.G., Morgan, I., Ghatei, M.A., *et al.* (2001). Evidence of an orexigenic role for cocaine- and amphetamine-regulated transcript after administration into discrete hypothalamic nuclei. Endocrinology *142*, 3457-3463.

Bewick, G.A., Kent, A., Campbell, D., Patterson, M., Ghatei, M.A., Bloom, S.R., and Gardiner, J.V. (2009). Mice with hyperghrelinemia are hyperphagic and glucose intolerant and have reduced leptin sensitivity. Diabetes *58*, 840-846.

Fray, P.J., Sahakian, B.J., Robbins, T.W., Koob, G.F., and Iversen, S.D. (1980). An observational method for quantifying the behavioural effects of dopamine agonists: contrasting effects of d-amphetamine and apomorphine. Psychopharmacology (Berl) *69*, 253-259.

Mystkowski, P., Shankland, E., Schreyer, S.A., LeBoeuf, R.C., Schwartz, R.S., Cummings, D.E., Kushmerick, M,, Schwartz, M.W. (2000). Validation of whole-body magnetic resonance spectroscopy as a tool to assess murine body composition. Int J Obes Relat Metab Disord 24, 719-24.

Pohlenz, J., Maqueem, A., Cua, K., Weiss, R.E., Van Sande, J., and Refetoff, S. (1999). Improved radioimmunoassay for measurement of mouse thyrotropin in serum: strain differences in thyrotropin concentration and thyrotroph sensitivity to thyroid hormone. Thyroid 9, 1265-1271.

Tschöp, M.H., Speakman, J.R., Arch, J.R., Auwerx, J., Brüning, J.C., Chan, L., Eckel, R.H., Farese, R.V. Jr, Galgani, J.E., Hambly, C., et al. (2011). A guide to analysis of mouse energy metabolism. Nat Methods. 9, 57-63.
