# Supplementary material for: Thyroid Hormone Receptor Beta in the Ventromedial Hypothalamus Is Essential for the Physiological Regulation of Food Intake and Body Weight
Source: Cell Rep. 2017 Jun 13;19(11):2202–9. doi: 10.1016/j.celrep.2017.05.066 (PMC5478879; doi:10.1016/j.celrep.2017.05.066)
Supplement: Document S2. Article plus Supplemental Information [file mmc4.pdf]

Cell Reports

Report

Thyroid Hormone Receptor Beta in the Ventromedial Hypothalamus Is Essential for the Physiological Regulation of Food Intake and Body Weight

Graphical Abstract

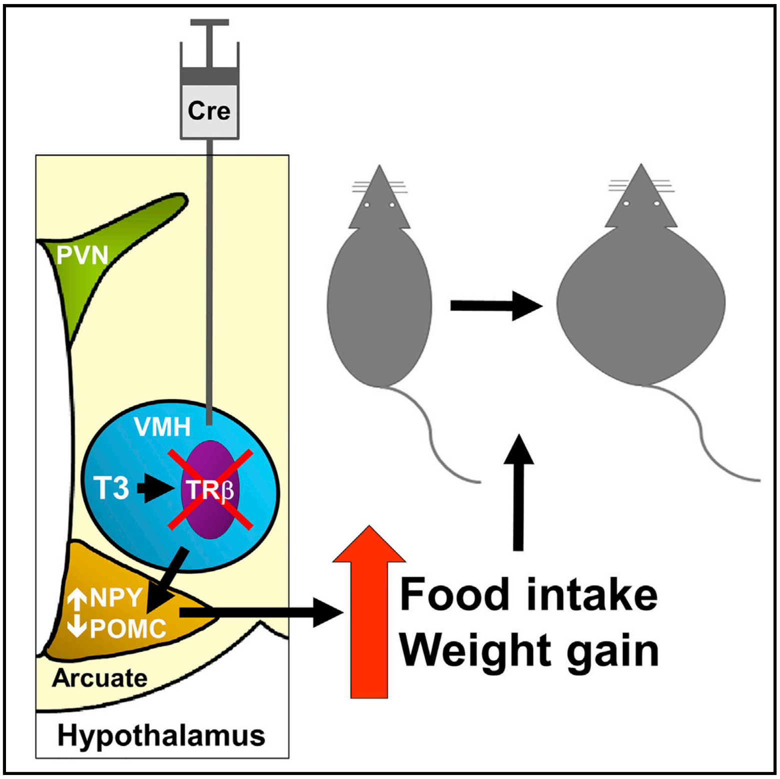

Authors

Saira Hameed, Michael Patterson, Waljit S. Dhillo, ..., J.H. Duncan Bassett, Graham R. Williams, James V. Gardiner

Correspondence

graham.williams@imperial.ac.uk (G.R.W.), j.gardiner@imperial.ac.uk (J.V.G.)

In Brief

Hameed et al. report that selective knockdown of a thyroid hormone receptor in the mouse hypothalamus results in a phenotype of severe obesity, overeating, and reduced energy expenditure, which may be due to downstream changes in the expression of hypothalamic regulators of food intake.

Highlights

- The ventromedial hypothalamus (VMH) expresses thyroid hormone receptor beta (TRβ)
- In mice, selective knockdown of TRβ (TRβ^{-}) in the VMH results in severe obesity
- The obesity is due to overeating (hyperphagia) and reduced energy expenditure
- TRβ^{-} mice have altered expression of hypothalamic regulators of food intake

Accession Numbers

GSE98690

Hameed et al., 2017, Cell Reports *19*, 2202–2209
June 13, 2017 © 2017 The Author(s).
http://dx.doi.org/10.1016/j.celrep.2017.05.066

CellPress

OPEN ACCESS
CellPress

Cell Reports
Report

Thyroid Hormone Receptor Beta in the Ventromedial Hypothalamus Is Essential for the Physiological Regulation of Food Intake and Body Weight

Saira Hameed,[1] Michael Patterson,[1,6] Waljit S. Dhillo,[1] Sofia A. Rahman,[2] Yue Ma,[1] Christopher Holton,[1] Apostolos Gogakos,[2] Giles S.H. Yeo,[3] Brian Y.H. Lam,[3] Joseph Polex-Wolf,[3] Wiebke Fenske,[1] Jimmy Bell,[4] Jelena Anastasovska,[4] Jacques Samarut,[5] Stephen R. Bloom,[1] J.H. Duncan Bassett,[2] Graham R. Williams,[2,*] and James V. Gardiner[1,7,*]

[1]Section of Investigative Medicine, Division of Diabetes, Endocrinology and Metabolism, Imperial College London, London W12 0NN, UK
[2]Molecular Endocrinology Laboratory, Hammersmith Campus, Imperial College London, London W12 0NN, UK
[3]University of Cambridge Metabolic Research Laboratories, Wellcome Trust-MRC Institute of Metabolic Science, Addenbrooke's Hospital, Cambridge CB2 0QQ, UK
[4]Metabolic and Molecular Imaging Group, Imperial College London, London W12 0NN, UK
[5]Institut de Génomique Fonctionnelle de Lyon, Ecole Normale Supérieure de Lyon, 69364 Lyon, France
[6]Department of Life Sciences, University of Roehampton, London SW15 4JD, UK
[7]Lead Contact
*Correspondence: graham.williams@imperial.ac.uk (G.R.W.), j.gardiner@imperial.ac.uk (J.V.G.)
http://dx.doi.org/10.1016/j.celrep.2017.05.066

SUMMARY

The obesity epidemic is a significant global health issue. Improved understanding of the mechanisms that regulate appetite and body weight will provide the rationale for the design of anti-obesity therapies. Thyroid hormones play a key role in metabolic homeostasis through their interaction with thyroid hormone receptors (TRs), which function as ligand-inducible transcription factors. The TR-beta isoform (TRβ) is expressed in the ventromedial hypothalamus (VMH), a brain area important for control of energy homeostasis. Here, we report that selective knockdown of TRβ in the VMH of adult mice results in severe obesity due to hyperphagia and reduced energy expenditure. The observed increase in body weight is of a similar magnitude to murine models of the most extreme forms of monogenic obesity. These data identify TRβ in the VMH as a major physiological regulator of food intake and energy homeostasis.

INTRODUCTION

Energy homeostasis is regulated by neurotransmitters and by humoral factors including thyroid hormones, which act within the hypothalamus and systemically to regulate food intake (Coppola et al., 2007; Coll et al., 2007) and energy expenditure (Kim, 2008). The effects of the active form of thyroid hormone, 3,5,3′-L-triiodothyronine (T3), are mediated by two thyroid hormone receptors (TRα and TRβ), encoded by *Thra* and *Thrb*, respectively (Brent, 2012).

Metabolic phenotypes have been described in mice and humans with TR mutations. Mice with heterozygous dominant-negative mutations of TRα display a variety of metabolic phenotypes ranging from hypermetabolism, hyperphagia, and resistance to diet-induced obesity (Sjögren et al., 2007) to increased visceral adiposity, hypophagia, and impaired cold-induced adaptive thermogenesis (Liu et al., 2003). The variation in described phenotypes is likely to be due to the differing actions of individual mutant receptors on wild-type TR function (Ortiga-Carvalho et al., 2014). Humans with heterozygous dominant-negative mutations of TRα (resistance to thyroid hormone α [RTHα]) may be overweight or obese with reduced energy expenditure (Bochukova et al., 2012; Moran et al., 2013, 2014). Humans with heterozygous dominant-negative mutations of TRβ have RTHβ, resulting in high levels of circulating thyroid hormones and thyroid-stimulating hormone (TSH) due to impaired negative feedback of the hypothalamic-pituitary-thyroid axis (Ortiga-Carvalho et al., 2014). Humans with RTHβ may be overweight and hyperphagic (Mitchell et al., 2010) despite features of hyperthyroidism such as tachycardia and raised energy expenditure due to T3 actions in TRα-responsive tissues. These extensive studies demonstrate that thyroid hormone is an essential regulator of food intake and energy expenditure. Despite this, clinical and global gene targeting studies cannot differentiate between the developmental and adult, or systemic and central, effects of thyroid hormones.

The ventromedial hypothalamus (VMH) is a critical region of the brain involved in energy homeostasis. TRβ is the predominant TR isoform expressed in the VMH (Cook et al., 1992; Barrett et al., 2007), and previous studies suggest that thyroid hormones acting in the VMH regulate both food intake (Kong et al., 2004) and energy expenditure (López et al., 2010). Thus, we hypothesize that, in the VMH, TRβ physiologically regulates food intake and body weight. To investigate this hypothesis directly, we used stereotaxic Cre-lox gene targeting to generate a VMH-specific model of TRβ knockdown in adult mice.

 © 2017 The Author(s).
This is an open access article under the CC BY license (http://creativecommons.org/licenses/by/4.0/).

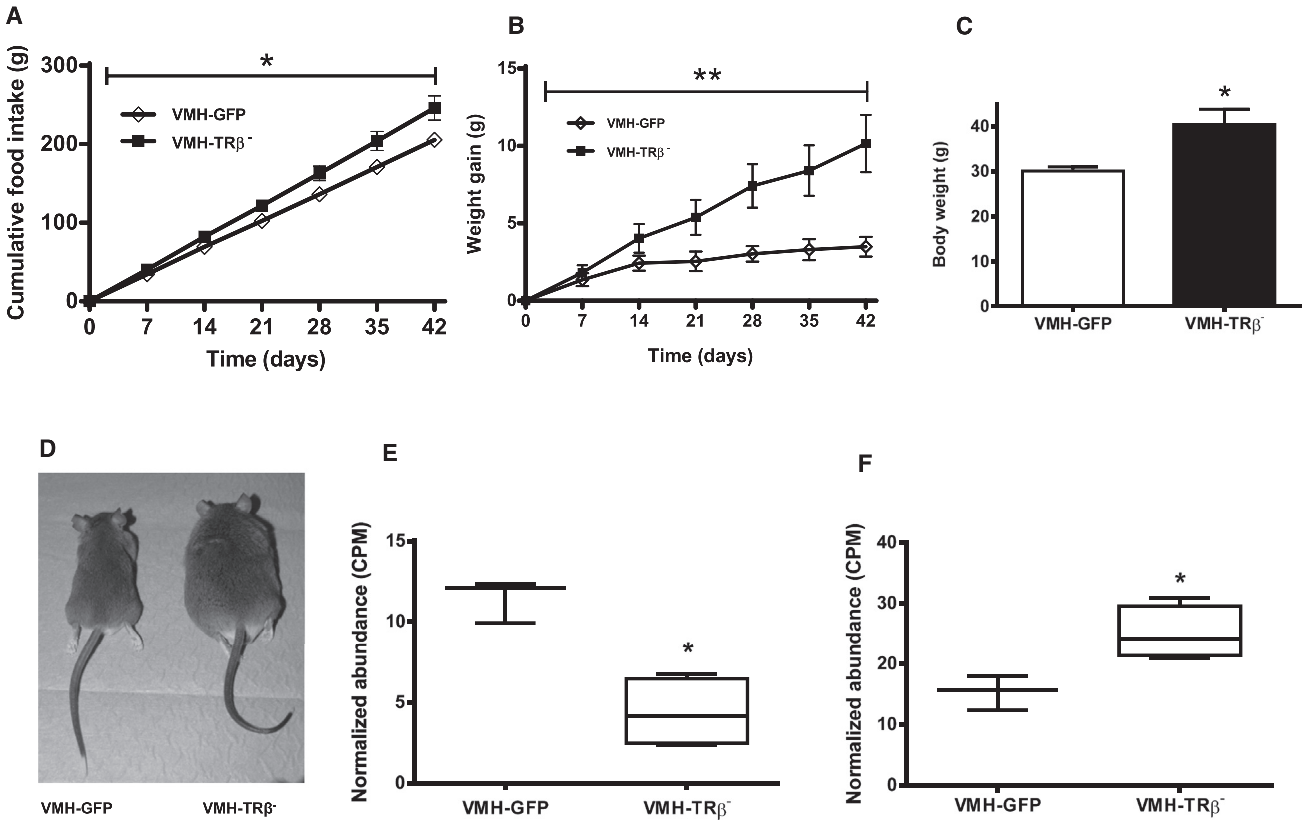

Figure 1. Effect of Reduced TRβ Expression in the VMH

(A) Cumulative food intake.

(B) Cumulative body weight change.

(C) Body weight on day 42.

(D) Photograph of VMH-GFP and VMH-TRβ$^-$ mouse.

(E) Hypothalamic expression of *Pomc*.

(F) Hypothalamic expression of *Npy*.

In (A)–(C), the results are mean ± SEM; n = 10 for VMH-GFP and 11 for VMH-TRβ$^-$. In (E) and (F), the results are median, and whiskers are minimum and maximum; n = 3 for VMH-GFP and 4 for VMH-TRβ$^-$; *$p < 0.05$; **$p < 0.01$. Food intake and body weight were analyzed using a generalized estimating equation exchangeable correlation matrix and robust SEs (GEE), body weight data t test. See also Figures S1–S3 and Tables S1 and S2.

RESULTS

Tissue-Specific Knockdown of TRβ in the VMH in Adult Mice

We knocked down TRβ in the VMH of adult male mice using Cre-mediated excision of a floxed critical exon in the *Thrb* gene. This approach enabled temporally and spatially controlled reduction of TRβ expression specifically in the VMH of adult mice. This model eliminates the developmental consequences and abnormal systemic thyroid hormone levels that occur in global TRβ mutant mice (Ortiga-Carvalho et al., 2014) or in hypothyroid and thyrotoxic animals (Ishii et al., 2003; López et al., 2010).

The *Thrb^{flox}* allele contains loxP sites flanking exon 5 of *Thrb* (Winter et al., 2009) (Figure S1A). *Cre-recombinase*-mediated excision of this critical exon results in inactivation of *Thrb* (Winter et al., 2009). *Cre recombinase* was introduced into the VMH of adult male *Thrb$^{flox/flox}$* mice by stereotaxic injection of recombinant adeno-associated virus (rAAV) expressing a Cre-GFP fusion protein to generate mice with reduced TRβ expression in the VMH (VMH-TRβ$^-$) mice. *Thrb$^{flox/flox}$* mice injected with rAAV encoding GFP into the VMH (VMH-GFP) were used as controls. Cre-mediated excision of the *Thrb*^{flox} allele was confirmed by PCR of DNA from whole hypothalami of VMH-TRβ$^-$ mice (Figure S1B). The *Thrb^{flox}* allele was not excised in either the cerebellum or brainstem, indicating rAAV did not enter the ventricular system following stereotaxic injection (Figure S1B). Fluorescence microscopy and in situ hybridization (ISH) both confirmed transgene expression localized to the VMH in both groups of mice (Figures S2A and S2B). ISH using a probe specific for the floxed exon of *Thrb* demonstrated reduced expression within the VMH of VMH-TRβ$^-$ mice compared with controls (Figures S2C and S2D).

Selective Knockdown of TRβ in the VMH in Adult Mice Results in Hyperphagia and Obesity

VMH-TRβ$^-$ mice consumed more food and gained more weight than controls (Figures 1A and 1B). Weight gain in VMH-TRβ$^-$

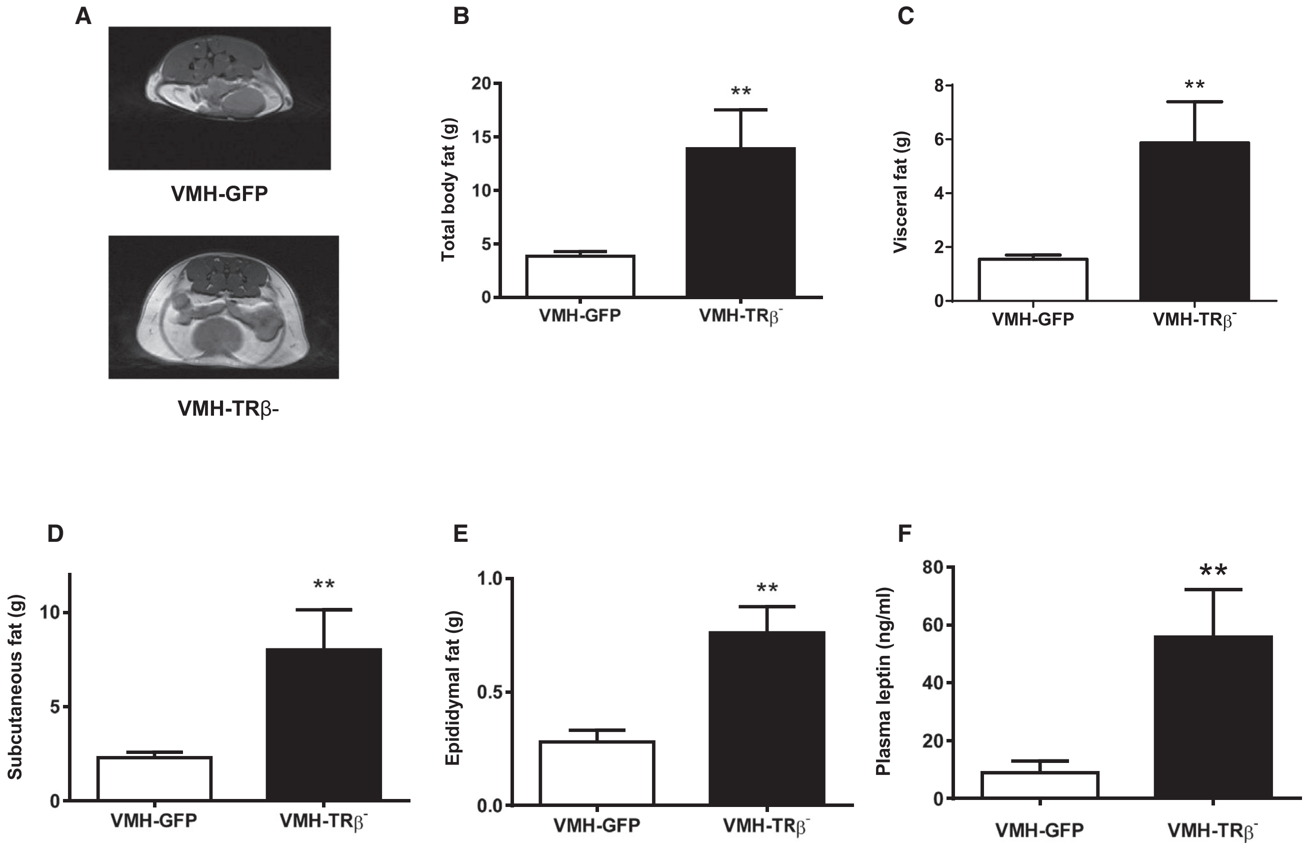

Figure 2. White Adipose Tissue Mass and Distribution

MRI quantification of fat demonstrated that VMH-TRβ^{-} mice had significantly higher fat mass.

(A) Representative transverse T1-weighted MR images through the abdominal region of a VMH-GFP and VMH-TRβ^{-} mouse.

(B) Total body fat.

(C) Visceral fat.

(D) Subcutaneous fat.

(E) Epididymal fat pad weight on day 42 (n = 10).

(F) Plasma leptin levels on day 42 (n = 10).

Results are mean ± SEM (n = 3 per group unless stated). **$p < 0.01$ versus control, t test with Bonferroni correction. See also Figure S4.

mice was three times greater than that of control mice by the end of the study (Figures 1C and 1D).

Whole hypothalami for RNA-sequencing (RNA-seq) analysis were collected from mice before significant changes in body weight had occurred. This was so that changes in expression are likely to be due to changes in thyroid hormone signaling rather than secondary effects of the increase in body weight and food intake. Differential expression analysis was performed (Table S1). Pathway analysis of differentially expressed genes revealed an over-representation of genes involved in dopamine, growth hormone, and leptin signaling pathways, as well as genes that are involved in neuronal activity regulation including long-term potentiation (LTP) and long-term depression (LTD); these results were qualitatively the same when the false discovery rate (FDR) for analysis was set between 0.001 and 0.1 (Table S2). Among the genes differentially expressed, *Pomc* expression was decreased (log Fc −1.38, p = 9.33 × 10^{-7}) (Figure 1E), whereas *Npy* expression was increased (log Fc 0.7, p = 9.42 × 10^{-6}) (Figure 1F), whereas that of *Thrb* was not altered at the level of the whole hypothalamus (Table S1). Expression of steroidogenic factor 1 (*Nr5a1*), and uncoupling protein-2 (*Ucp2*), both of which are implicated in hypothalamic control of energy homeostasis (Majdic et al., 2002; Coppola et al., 2007), were unchanged. The differentially expressed genes were compared to those previously reported to be T3 responsive or directly regulated by T3 in cerebrocortical cells (Tables S1 and S2 and Figure S3) (Gil-Ibañez et al., 2017). Of the genes directly regulated by T3 in cerebrocortical cells, we identified 89 (~15%) were also significantly changed in our samples, among which was hairless (*Hr*). For genes regulated indirectly by T3, we identified 133 that were also changed (~9%).

Total, visceral, subcutaneous, and epididymal fat mass were all increased in VMH-TRβ^{-} mice compared to controls (Figures 2A–2E). In keeping with the increased adiposity, VMH-TRβ^{-} mice had a higher plasma leptin concentration than controls (Figure 2F).

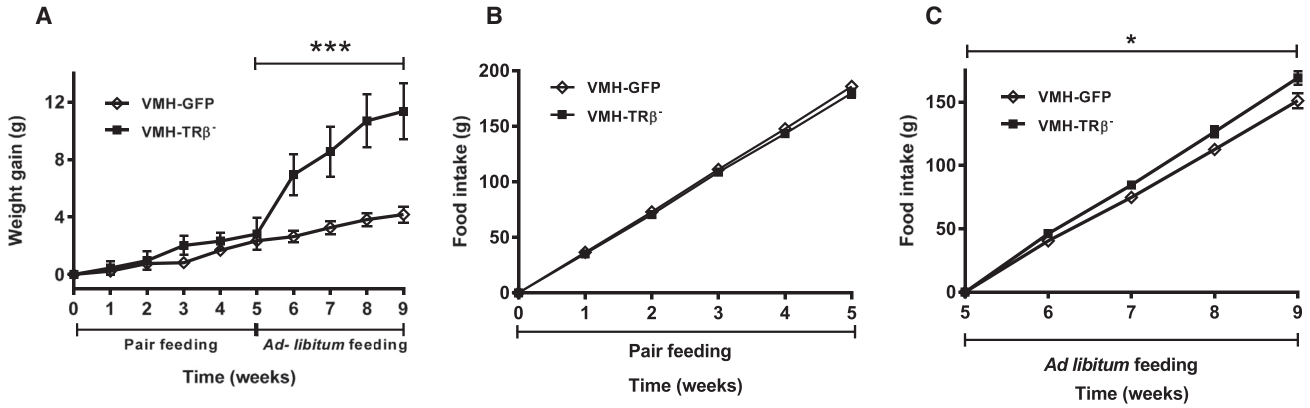

Figure 3. Effect of Pair-Feeding on VMH-TRβ⁻ Mice

(A) Weight gain over the entire period of the experiment. During weeks 0–5, food intake of each VMH-TRβ⁻ mouse was limited to that of a weight-matched, VMH-GFP littermate. From weeks 5 to 9, ad libitum access to food was restored.

(B) Food intake during the pair-feeding period.

(C) Food intake during the ad libitum feeding period.

Results are mean ± SEM. n = 9; GEE, *$p < 0.05$; ***$p < 0.001$.

VMH-TRβ⁻ Mice Are Systemically Euthyroid

Alterations in circulating thyroid hormones affect food intake and body weight (Pijl et al., 2001). Measurement of plasma TSH, thyroxine (T4), and T3 confirmed that both VMH-TRβ⁻ and control mice were euthyroid (Figures S4A–S4C).

VMH-TRβ⁻ Mice Are Insulin Resistant but Do Not Show Changes in the Expression of Genes Involved in Hypothalamic Glucose Sensing

Obese VMH-TRβ⁻ mice had high levels of fasting insulin (Figure S4D), as expected. However, when glucose tolerance and insulin tolerance were tested before the development of obesity in the VMH-TRβ⁻ mice, there were no differences between the VMH-TRβ⁻ and VMH-GFP mice (Figures S4E and S4F). RNA-seq analysis did not identify changes in expression of hypothalamic glucose-sensing genes.

Obesity in VMH-TRβ⁻ Mice Is Not Due to TRβ Knockdown in Other Brain Areas

To confirm that the observed weight gain and hyperphagia in VMH-TRβ⁻ mice resulted from reduced TRβ expression in the VMH and not spread through the ventricular system into other brain regions, rAAV-Cre was injected into both lateral ventricles of *Thrb*$^{flox/flox}$ mice; a control group of mice were injected with rAAV-GFP. There was no difference in cumulative food intake or body weight gain between these two groups (Figures S4G and S4H).

VMH-TRβ⁻ Mice Fail to Mount an Orexigenic Response to Administered T3

In order to validate loss of T3 signaling following TRβ inactivation in the VMH, we administered T3 to VMH-TRβ⁻ and VMH-GFP mice by subcutaneous injection. Over the 24-hr study period, T3 significantly increased food intake in VMH-GFP mice but VMH-TRβ⁻ mice failed to mount an orexigenic response to the administered T3 (Figure S4I).

VMH-TRβ⁻ Mice Do Not Become Obese When Pair-Fed to the Food Intake of Lean Controls

To investigate whether the hyperphagia contributed to, or was a consequence of, the development of the obese phenotype, VMH-TRβ⁻ mice were pair-fed to the food intake of a weight-matched VMH-GFP littermate for 5 weeks. During pair-feeding, there was no difference in cumulative body weight change or food intake (Figures 3A and 3B) or locomotor activity between the two groups.

After 5 weeks of pair-feeding, ad libitum access to food was restored for 4 weeks. Following restoration of free feeding, VMH-TRβ⁻ mice gained significantly more weight and consumed significantly more food than controls (Figures 3A and 3C).

VMH-TRβ⁻ Mice Have Reduced Energy Expenditure and Reduced Locomotor Activity

The contribution of changes in energy expenditure to the obese phenotype was investigated. Oxygen consumption (VO_2), carbon dioxide production (VCO_2), and locomotor activity were all decreased during the dark phase in ad libitum-fed VMH-TRβ⁻ mice both before and after the onset of obesity (Figures 4A–4C). By contrast, there was no difference in VO_2, VCO_2, or locomotor activity during the light phase (Figures 4A–4C). The decrease in nocturnal locomotion in VMH-TRβ⁻ mice was confirmed by behavioral analysis (Table S3). There was no difference in respiratory exchange ratio (RER) (Figure 4D) and no difference in brown adipose tissue (BAT) uncoupling protein-1 (*Ucp1*) expression (Figure 4E) between VMH-TRβ⁻ and control mice. In addition, VMH-TRβ⁻ mice have a normal body temperature (Figure S4J).

DISCUSSION

These studies identify hypothalamic TRβ as an important physiological regulator of appetite and body weight. Reduced TRβ expression in the VMH resulted in marked weight gain,

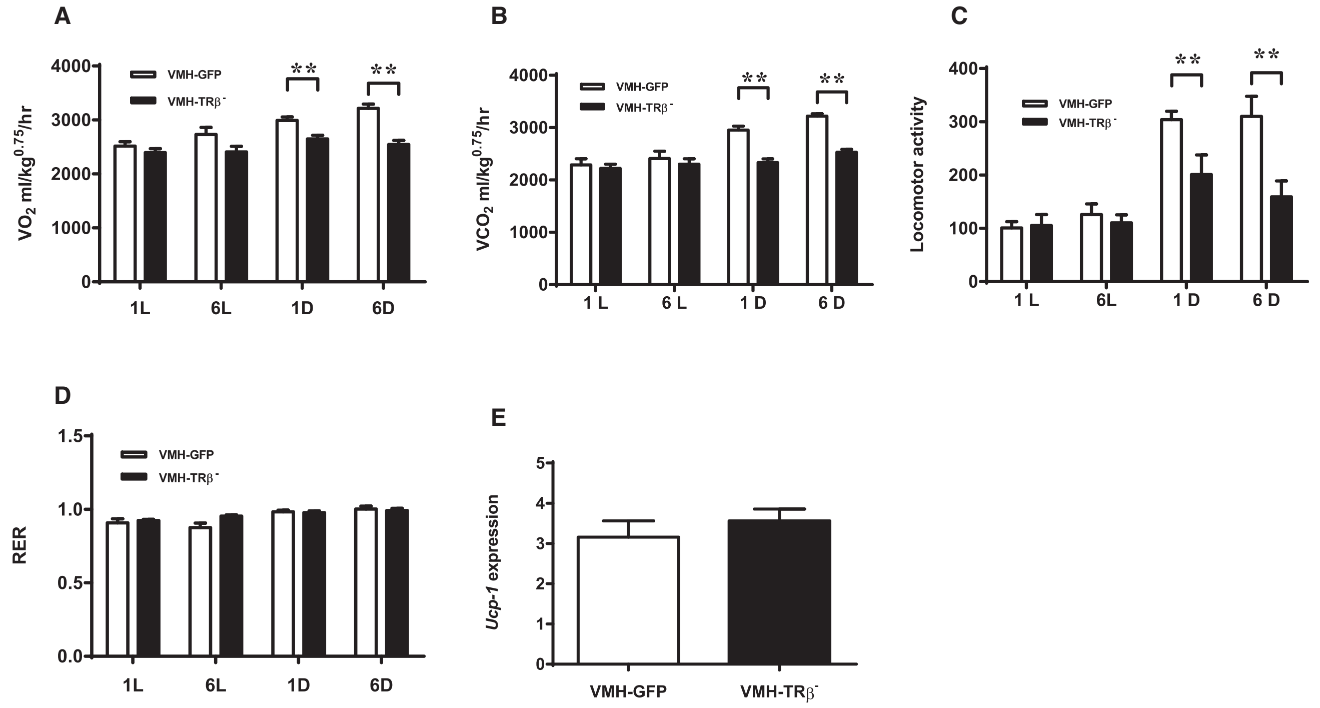

Figure 4. Energy Expenditure and Locomotor Activity in Mice with Reduced Expression of TRβ in the VMH

(A) Oxygen consumption.

(B) Carbon dioxide production.

(C) Locomotor activity.

(D) RER.

(E) *Ucp1* expression in BAT (n = 7 VMH-GFP and 11 VMH-TRβ⁻).

L, light phase; D, dark phase; 1, 1 week, and 6, 6 weeks, after recovery. Data are mean ± SEM (n = 5 VMH-GFP; n = 6 VMH-TRβ⁻); ANOVA with Student-Newman-Keuls analysis, **p < 0.01. See also Table S3.

comparable to severe forms of monogenic obesity (Tecott et al., 1995; Yaswen et al., 1999). The weight gain was a consequence of increased total body fat, and in particular a marked increase in subcutaneous and visceral white adipose tissue, the latter being an important risk factor for cardiovascular disease and diabetes (Montague and O'Rahilly, 2000).

VMH-TRβ⁻ mice ate more than control animals, and pair-feeding studies indicated that hyperphagia contributed directly to the obesity. Thus, VMH-TRβ⁻ mice remained lean when food intake was restricted but rapidly became obese when ad libitum feeding was restored.

Selective TRβ knockdown specifically in the VMH was confirmed by ISH and fluorescence microscopy. Although expression of *Thrb* was not reduced in the RNA-seq analysis, these samples are derived from whole hypothalami, and therefore the decrease in the level in the VMH is likely masked by the expression of *Thrb* throughout the rest of the sample. Indeed, the loss of TRβ function in the VMH was demonstrated by the failure of the expected orexigenic response to administered T3 in VMH-TRβ⁻ mice and further supported by the appropriate changes in genes directly regulated by T3. The possibility of the phenotype arising through virus spread to other CNS areas was excluded by rAAV-Cre injection into the lateral ventricles, which did not result in hyperphagia or obesity.

Previous work in rats has reported the acute orexigenic effect of exogenously administered T3 (Kong et al., 2004). Here, we show the endogenous effect of thyroid hormone action following selective TRβ knockdown. We suggest that our current work describes a local circuit within the VMH that physiologically regulates food intake as distinct from the feeding response to administered pharmacological doses of T3 analogous to the contrasting effects of NPY and PYY.

To investigate the underlying cause of hyperphagia in VMH-TRβ⁻ mice, hypothalamic gene expression patterns were determined by RNA-seq. The expression of *Pomc* and *Fto* were downregulated in the hypothalamus, whereas *Npy* was upregulated. POMC and FTO are thought to inhibit food intake, whereas NPY simulates food intake; therefore, these changes in expression may explain in part the phenotype observed.

Energy expenditure in VMH-TRβ⁻ mice was reduced both before and after the onset of obesity. There was no difference in BAT *Ucp1* expression between VMH-TRβ⁻ and control mice, suggesting that adaptive thermogenesis was unaffected. It is likely that changes in energy expenditure in VMH-TRβ⁻ mice resulted from decreased locomotor activity. The reduced locomotor activity is not a consequence of the obesity because it occurred before differences in body weight. In addition, during pair-feeding studies, the reduction in locomotor activity was lost,

possibly due to food-seeking behavior. This is likely to explain why body weight gain did not differ between the two groups before the restoration of ad libitum feeding. The energy expenditure and pair-feeding data indicate that both increased food intake and reduced locomotor activity contribute to obesity in VMH-TRβ^{-} mice.

In contrast to VMH-TRβ^{-} mice, global heterozygous TRβ-knockout mice do not have an obese phenotype (Ortiga-Carvalho et al., 2014). This may be explained by the peripheral hyperthyroidism of these mice. In addition, the appetite circuits within the hypothalamus are subject to developmental plasticity and compensatory redundancy (Bouret et al., 2004; Horvath, 2005). For example, neither global deletion of *Agrp* and/or *Npy* nor ablation of arcuate AgRP/NPY neurons in neonatal mice results in a metabolic phenotype (Erickson et al., 1996; Qian et al., 2002; Luquet et al., 2005), whereas ablation of these neurons in adult mice produces profound hypophagia and starvation (Luquet et al., 2005; Gardiner et al., 2005; Bewick et al., 2005). Similar developmental compensation may occur in global TRβ-knockout mice.

Studies using adenovirus-mediated expression of a dominant-negative TR (DN-TR) in the rat VMH have been reported (López et al., 2010). Although VMH DN-TR expression did not affect food intake or body weight in euthyroid animals, it prevented weight loss in thyrotoxic rats and resulted in reduced hypothalamic AMP-activated protein kinase (AMPK) expression (López et al., 2010). AMPK expression was unchanged in our model. DN-TR interferes with the actions of both TRα and TRβ and exerts a marked repressive effect on gene transcription (Ortiga-Carvalho et al., 2014; Ferrara et al., 2012). By contrast, VMH-TRβ^{-} mice have only reduced TRβ activity rather than the pathological repression of TR target genes that is present in animals expressing a dominant-negative receptor. This fundamental difference is likely to explain the contrasting phenotypes observed in these two models.

In summary, we have shown that hypothalamic TRβ is an important physiological regulator of energy homeostasis because TRβ knockdown in the VMH results in a phenotype of hyperphagia and severe obesity that is comparable to some of the most extreme forms of monogenic obesity (Tecott et al., 1995; Yaswen et al., 1999). Our findings provide insights into the central regulation of energy homeostasis by TRβ that could be a target for anti-obesity therapies.

EXPERIMENTAL PROCEDURES

Animals

Thrb$^{flox/flox}$ mice (Winter et al., 2009) were genotyped by PCR using specific oligonucleotide primers (Figure S1). Mice were housed in single cages and maintained under a controlled environment (temperature, 21–23°C; 12-h light–dark cycle, lights on at 07:00) with ad libitum access to chow and water (RM1; SDS Diets), except where stated. Male mice that were 8 weeks old at the start of procedures were used in all experiments. All animal studies were approved under the Animals (Scientific Procedures) Act (1986) (Project License Number 70_7229) and approved by the Animal Welfare and Ethical Review Body, Imperial College London, which is signed up to the ARRIVE (Animal Research: Reporting of In Vivo Experiments) guidelines.

rAAV Preparation

rAAV was produced (Grimm et al., 1998) and isolated (Zolotukhin et al., 1999), as previously described.

Confirmation of rAAV Transgene Expression, *Thrb* Excision, and Reduced TRβ Expression in the VMH

Excision of the *Thrb*^{flox} allele within the hypothalamus was confirmed by PCR (Figure S1). ISH using a probe specific to the excised portion of TRβ was performed to confirm reduced TRβ expression within the VMH (Smith et al., 2008).

Measurement of Energy Expenditure

Metabolic parameters were measured by indirect calorimetry using an open-circuit Oxymax system of the Comprehensive Lab Animal Monitoring System (Columbus Instruments) (Gardiner et al., 2010).

RNA-Seq Analysis

RNA-seq analysis was performed using hypothalamic RNA from VMH-GFP (n = 3) and VMH-TRβ^{-} (n = 4) mice using next-generation sequencing (NGS) technologies (Imperial BRC Genomics Laboratory, Imperial College London). For further details, see Supplemental Experimental Procedures.

Statistical Analyses

Cumulative food intake and body weight data were analyzed using generalized estimating equations with exchangeable correlation matrix and robust SEs. Differences between two groups at individual time points were analyzed by unpaired t tests; for multiple comparisons, a Bonferroni correction was applied. Values from the behavioral study were analyzed using a one-way ANOVA followed by Kruskal-Wallis test. Data from the energy expenditure test were analyzed using a one-way ANOVA followed by a Newman-Keuls test. Plasma thyroid hormones were compared using Mann-Whitney U test. Differences between groups were considered statistically significant at the 95% confidence level ($p < 0.05$).

ACCESSION NUMBERS

The accession number for the RNA-seq data reported in this paper is GEO: GSE98690.

SUPPLEMENTAL INFORMATION

Supplemental Information includes Supplemental Experimental Procedures, four figures, and three tables and can be found with this article online at http://dx.doi.org/10.1016/j.celrep.2017.05.066.

AUTHOR CONTRIBUTIONS

W.S.D., S.R.B., J.H.D.B., G.R.W., and J.V.G. conceived of and supervised the project. S.H., M.P., W.S.D., S.A.R., Y.M., C.H., W.F., and J.V.G. conducted the majority of the experiments. S.A.R. and A.G. maintained the mice. A.G. and J.H.D.B. prepared the TRβ probe. J.B. and J.A. performed the MRI study. G.S.H.Y., B.Y.H.L., and J.P.-W. performed the RNA-seq experiments and analysis. J.S. generated the *Thrb*$^{flox/flox}$ mice. S.H., W.S.D., S.R.B., J.H.D.B., G.R.W., and J.V.G. wrote the manuscript. All authors discussed the results and commented on the manuscript.

ACKNOWLEDGMENTS

We thank Dr. Roy Weiss, Dr. Samuel Refetoff, and Dr. XiaoHui Liao (University of Chicago) for measurement of tT3, tT4, and TSH concentrations. This work is funded by BBSRC BB/F021704. Investigative Medicine is funded by grants from MRC, BBSRC, and NIHR, by an FP7-HEALTH-2009-241592 EuroCHIP grant, and by the NIHR Imperial Biomedical Research Centre. S.H. receives a MRC CRTF, an NIHR CL, and a Society for Endocrinology ECG. W.S.D. is funded by an NIHR Research Professorship. G.R.W. and J.H.D.B. are supported by a Wellcome Trust Strategic Award (101123/Z/13/A), Wellcome Trust Investigator Award (110141/Z/15/Z and 110140/Z/15/Z), EU HORIZON 2020 Grant (THYRAGE-666869), and MRC funding (MR/N01121X/1). This work used the computing resources of the UK MEDical BIOinformatics partnership—aggregation, integration, visualization, and analysis of large, complex data (UK MED-BIO)—which is supported by the MRC (Grant Number

MR/L01632X/1). University of Cambridge Metabolic Research Laboratories are supported by the UK Medical Research Council (MRC) Metabolic Disease Unit (MRC_MC_UU_12012/1 and MRC_MC_UU_12012/5), a Wellcome Trust Strategic Award (100574/Z/12/Z), and the Helmholtz Alliance ICEMED.

Received: May 28, 2015
Revised: April 6, 2017
Accepted: May 19, 2017
Published: June 13, 2017

REFERENCES

Barrett, P., Ebling, F.J., Schuhler, S., Wilson, D., Ross, A.W., Warner, A., Jethwa, P., Boelen, A., Visser, T.J., Ozanne, D.M., et al. (2007). Hypothalamic thyroid hormone catabolism acts as a gatekeeper for the seasonal control of body weight and reproduction. Endocrinology *148*, 3608–3617.

Bewick, G.A., Gardiner, J.V., Dhillo, W.S., Kent, A.S., White, N.E., Webster, Z., Ghatei, M.A., and Bloom, S.R. (2005). Post-embryonic ablation of AgRP neurons in mice leads to a lean, hypophagic phenotype. FASEB J. *19*, 1680–1682.

Bochukova, E., Schoenmakers, N., Agostini, M., Schoenmakers, E., Rajanayagam, O., Keogh, J.M., Henning, E., Reinemund, J., Gevers, E., Sarri, M., et al. (2012). A mutation in the thyroid hormone receptor alpha gene. N. Engl. J. Med. *366*, 243–249.

Bouret, S.G., Draper, S.J., and Simerly, R.B. (2004). Trophic action of leptin on hypothalamic neurons that regulate feeding. Science *304*, 108–110.

Brent, G.A. (2012). Mechanisms of thyroid hormone action. J. Clin. Invest. *122*, 3035–3043.

Coll, A.P., Farooqi, I.S., and O'Rahilly, S. (2007). The hormonal control of food intake. Cell *129*, 251–262.

Cook, C.B., Kakucska, I., Lechan, R.M., and Koenig, R.J. (1992). Expression of thyroid hormone receptor beta 2 in rat hypothalamus. Endocrinology *130*, 1077–1079.

Coppola, A., Liu, Z.W., Andrews, Z.B., Paradis, E., Roy, M.C., Friedman, J.M., Ricquier, D., Richard, D., Horvath, T.L., Gao, X.B., and Diano, S. (2007). A central thermogenic-like mechanism in feeding regulation: an interplay between arcuate nucleus T3 and UCP2. Cell Metab. *5*, 21–33.

Erickson, J.C., Clegg, K.E., and Palmiter, R.D. (1996). Sensitivity to leptin and susceptibility to seizures of mice lacking neuropeptide Y. Nature *381*, 415–421.

Ferrara, A.M., Onigata, K., Ercan, O., Woodhead, H., Weiss, R.E., and Refetoff, S. (2012). Homozygous thyroid hormone receptor β-gene mutations in resistance to thyroid hormone: three new cases and review of the literature. J. Clin. Endocrinol. Metab. *97*, 1328–1336.

Gardiner, J.V., Kong, W.M., Ward, H., Murphy, K.G., Dhillo, W.S., and Bloom, S.R. (2005). AAV mediated expression of anti-sense neuropeptide Y cRNA in the arcuate nucleus of rats results in decreased weight gain and food intake. Biochem. Biophys. Res. Commun. *327*, 1088–1093.

Gardiner, J.V., Bataveljic, A., Patel, N.A., Bewick, G.A., Roy, D., Campbell, D., Greenwood, H.C., Murphy, K.G., Hameed, S., Jethwa, P.H., et al. (2010). Prokineticin 2 is a hypothalamic neuropeptide that potently inhibits food intake. Diabetes *59*, 397–406.

Gil-Ibañez, P., García-García, F., Dopazo, J., Bernal, J., and Morte, B. (2017). Global transcriptome analysis of primary cerebrocortical cells: identification of genes regulated by triiodothyronine in specific cell types. Cereb. Cortex *27*, 706–717.

Grimm, D., Kern, A., Rittner, K., and Kleinschmidt, J.A. (1998). Novel tools for production and purification of recombinant adenoassociated virus vectors. Hum. Gene Ther. *9*, 2745–2760.

Horvath, T.L. (2005). The hardship of obesity: a soft-wired hypothalamus. Nat. Neurosci. *8*, 561–565.

Ishii, S., Kamegai, J., Tamura, H., Shimizu, T., Sugihara, H., and Oikawa, S. (2003). Hypothalamic neuropeptide Y/Y1 receptor pathway activated by a reduction in circulating leptin, but not by an increase in circulating ghrelin, contributes to hyperphagia associated with triiodothyronine-induced thyrotoxicosis. Neuroendocrinology *78*, 321–330.

Kim, B. (2008). Thyroid hormone as a determinant of energy expenditure and the basal metabolic rate. Thyroid *18*, 141–144.

Kong, W.M., Martin, N.M., Smith, K.L., Gardiner, J.V., Connoley, I.P., Stephens, D.A., Dhillo, W.S., Ghatei, M.A., Small, C.J., and Bloom, S.R. (2004). Triiodothyronine stimulates food intake via the hypothalamic ventromedial nucleus independent of changes in energy expenditure. Endocrinology *145*, 5252–5258.

Liu, Y.Y., Schultz, J.J., and Brent, G.A. (2003). A thyroid hormone receptor alpha gene mutation (P398H) is associated with visceral adiposity and impaired catecholamine-stimulated lipolysis in mice. J. Biol. Chem. *278*, 38913–38920.

López, M., Varela, L., Vázquez, M.J., Rodríguez-Cuenca, S., González, C.R., Velagapudi, V.R., Morgan, D.A., Schoenmakers, E., Agassandian, K., Lage, R., et al. (2010). Hypothalamic AMPK and fatty acid metabolism mediate thyroid regulation of energy balance. Nat. Med. *16*, 1001–1008.

Luquet, S., Perez, F.A., Hnasko, T.S., and Palmiter, R.D. (2005). NPY/AgRP neurons are essential for feeding in adult mice but can be ablated in neonates. Science *310*, 683–685.

Majdic, G., Young, M., Gomez-Sanchez, E., Anderson, P., Szczepaniak, L.S., Dobbins, R.L., McGarry, J.D., and Parker, K.L. (2002). Knockout mice lacking steroidogenic factor 1 are a novel genetic model of hypothalamic obesity. Endocrinology *143*, 607–614.

Mitchell, C.S., Savage, D.B., Dufour, S., Schoenmakers, N., Murgatroyd, P., Befroy, D., Halsall, D., Northcott, S., Raymond-Barker, P., Curran, S., et al. (2010). Resistance to thyroid hormone is associated with raised energy expenditure, muscle mitochondrial uncoupling, and hyperphagia. J. Clin. Invest. *120*, 1345–1354.

Montague, C.T., and O'Rahilly, S. (2000). The perils of portliness: causes and consequences of visceral adiposity. Diabetes *49*, 883–888.

Moran, C., Schoenmakers, N., Agostini, M., Schoenmakers, E., Offiah, A., Kydd, A., Kahaly, G., Mohr-Kahaly, S., Rajanayagam, O., Lyons, G., et al. (2013). An adult female with resistance to thyroid hormone mediated by defective thyroid hormone receptor α. J. Clin. Endocrinol. Metab. *98*, 4254–4261.

Moran, C., Agostini, M., Visser, W.E., Schoenmakers, E., Schoenmakers, N., Offiah, A.C., Poole, K., Rajanayagam, O., Lyons, G., Halsall, D., et al. (2014). Resistance to thyroid hormone caused by a mutation in thyroid hormone receptor (TR)α1 and TRα2: clinical, biochemical, and genetic analyses of three related patients. Lancet Diabetes Endocrinol. *2*, 619–626.

Ortiga-Carvalho, T.M., Sidhaye, A.R., and Wondisford, F.E. (2014). Thyroid hormone receptors and resistance to thyroid hormone disorders. Nat. Rev. Endocrinol. *10*, 582–591.

Pijl, H., de Meijer, P.H., Langius, J., Coenegracht, C.I., van den Berk, A.H., Chandie Shaw, P.K., Boom, H., Schoemaker, R.C., Cohen, A.F., Burggraaf, J., and Meinders, A.E. (2001). Food choice in hyperthyroidism: potential influence of the autonomic nervous system and brain serotonin precursor availability. J. Clin. Endocrinol. Metab. *86*, 5848–5853.

Qian, S., Chen, H., Weingarth, D., Trumbauer, M.E., Novi, D.E., Guan, X., Yu, H., Shen, Z., Feng, Y., Frazier, E., et al. (2002). Neither agouti-related protein nor neuropeptide Y is critically required for the regulation of energy homeostasis in mice. Mol. Cell. Biol. *22*, 5027–5035.

Sjögren, M., Alkemade, A., Mittag, J., Nordström, K., Katz, A., Rozell, B., Westerblad, H., Arner, A., and Vennström, B. (2007). Hypermetabolism in mice caused by the central action of an unliganded thyroid hormone receptor alpha1. EMBO J. *26*, 4535–4545.

Smith, K.L., Gardiner, J.V., Ward, H.L., Kong, W.M., Murphy, K.G., Martin, N.M., Ghatei, M.A., and Bloom, S.R. (2008). Overexpression of CART in the PVN increases food intake and weight gain in rats. Obesity (Silver Spring) *16*, 2239–2244.

Tecott, L.H., Sun, L.M., Akana, S.F., Strack, A.M., Lowenstein, D.H., Dallman, M.F., and Julius, D. (1995). Eating disorder and epilepsy in mice lacking 5-HT2c serotonin receptors. Nature *374*, 542–546.

Winter, H., Rüttiger, L., Müller, M., Kuhn, S., Brandt, N., Zimmermann, U., Hirt, B., Bress, A., Sausbier, M., Conscience, A., et al. (2009). Deafness in TRbeta mutants is caused by malformation of the tectorial membrane. J. Neurosci. *29*, 2581–2587.

Yaswen, L., Diehl, N., Brennan, M.B., and Hochgeschwender, U. (1999). Obesity in the mouse model of pro-opiomelanocortin deficiency responds to peripheral melanocortin. Nat. Med. *5*, 1066–1070.

Zolotukhin, S., Byrne, B.J., Mason, E., Zolotukhin, I., Potter, M., Chesnut, K., Summerford, C., Samulski, R.J., and Muzyczka, N. (1999). Recombinant adeno-associated virus purification using novel methods improves infectious titer and yield. Gene Ther. *6*, 973–985.

Cell Reports, Volume *19*

Supplemental Information

Thyroid Hormone Receptor Beta in the Ventromedial Hypothalamus Is Essential for the Physiological Regulation of Food Intake and Body Weight

Saira Hameed, Michael Patterson, Waljit S. Dhillo, Sofia A. Rahman, Yue Ma, Christopher Holton, Apostolos Gogakos, Giles S.H. Yeo, Brian Y.H. Lam, Joseph Polex-Wolf, Wiebke Fenske, Jimmy Bell, Jelena Anastasovska, Jacques Samarut, Stephen R. Bloom, J.H. Duncan Bassett, Graham R. Williams, and James V. Gardiner

A

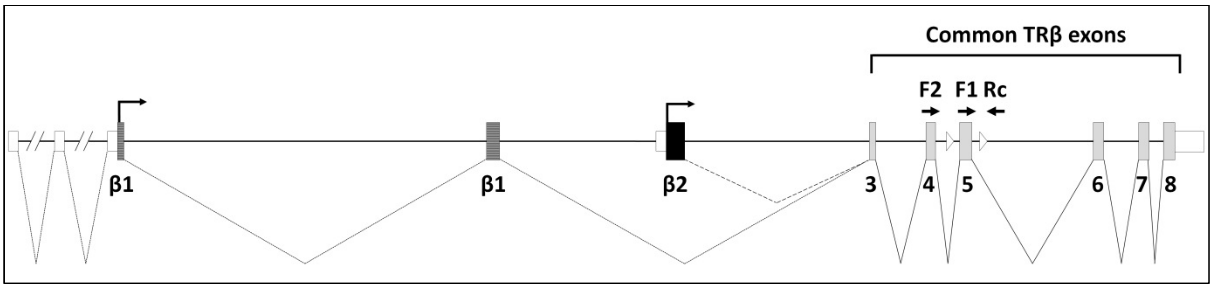

B

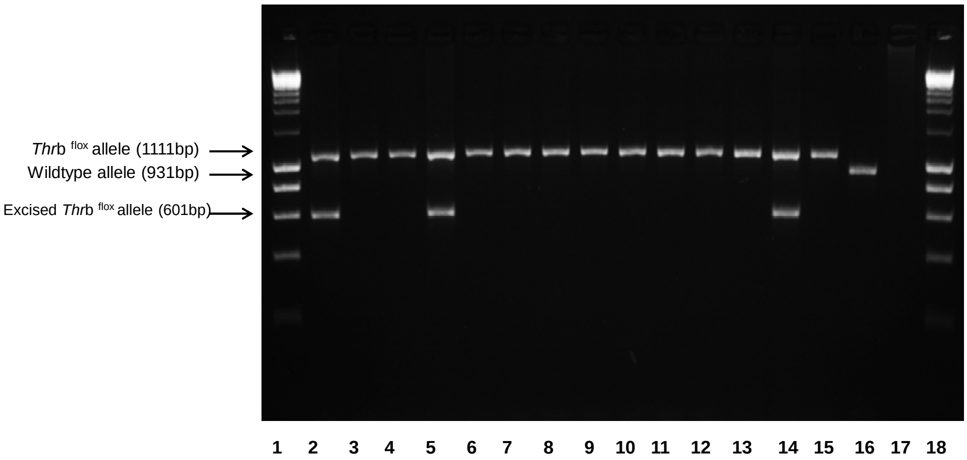

Supplemental Data Items

Figure S1 Schematic representation of the *Thrb*^{flox} allele and verification of excision of the *Thrb*^{flox} allele related to figure 1

A) Genomic structure of *Thrb* (NC_000080.6 (17660960-18038088)) showing the locations of the thyroid hormone receptor beta 1 (*TRβ1:* NM_001113417.1) and beta 2 (*TRβ2:* NM_009380.3) isoforms. White boxes represent untranslated exons, shaded boxes indicate unique 5’ *Thrβ1* exons, the black box shows the unique 5’ *Thrβ2* exon, and the light grey boxes show the 6 exons common to both isoforms. The positions of the two LoxP sites flanking exon 5 are indicated by the white triangles. The wild type and *Thrb*^{flox} alleles were amplified using the forward (F1: 5’-CAGCCACTGGAAGCAGAAG-3’) and reverse primers (Rc: 5’-AACGTCACTGTTGTGGTGTACAGG-3’). PCR amplification of the wild type allele resulted in a 931bp product whereas the product of the *Thrb*^{flox} allele was 1111bp in size. The excised *Thrb*^{flox} allele was amplified using the forward primer (F2: 5’-CATCTATGTTGGCATGGCAACAGACT-3’) and reverse primer (Rc) the resulting product being 601bp in size.

B) Agarose gel visualized under UV illumination of PCR on DNA to demonstrate excision of the *Thrb*^{flox} allele in *Thrb*$^{flox/flox}$ mice, restricted to the hypothalamus, following intra-VMH injection of rAAV-Cre. Arrows donate position of *Thrb*^{flox} allele (1111bp), wildtype allele (931bp) and the excised *Thrb*^{flox} allele (601bp). Lane 1: HyperLadder I™ (DNA molecular weight marker). Lanes 2 and 5: hypothalamic DNA from two *Thrb*$^{flox/flox}$ mice injected with rAAV-Cre into the VMH, denoted as VMH-$TR\beta^-$ mouse 1 and VMH-$TR\beta^-$ mouse 2 respectively, each showing a band at 1111bp representing the *Thrb*^{flox} allele and a band at 601bp representing the excised *Thrb*$^{\Delta VMH}$ allele. Lanes 3 and 6: PCR performed on DNA extracted from the cerebellum (lane 3, VMH-$TR\beta^-$ mouse 1; lane 6, VMH-$TR\beta^-$ mouse 2). Lanes 4 and 7: PCR performed on DNA extracted from and brainstem (lane 4, VMH-$TR\beta^-$ mouse 1; lane 7, VMH-$TR\beta^-$ mouse 2). In these lanes only the band at 1111bp representing the *Thrb*^{flox} allele is present. The absence of a band at 601bp in these lanes demonstrates that the *Thrb*^{flox} allele has not been excised in these extra-hypothalamic brain tissues. Lanes 8 and 11: hypothalamic DNA from two *Thrb*$^{flox\ /flox}$ mice injected with rAAV-GFP into the VMH, denoted as VMH-GFP-mouse 1 and VMH-GFP-mouse 2 respectively, each showing a band at 1111bp representing the *Thrb*^{flox} allele. The absence of a band at 601bp in these lanes demonstrates that the *Thrb*^{flox} allele has not been excised in the hypothalami of the rAAV-GFP injected mice. Lanes 9 and 12: PCR performed on DNA extracted from the cerebellum (lane 9, VMH-GFP-1-mouse 1; lane 12, VMH-GFP-mouse 2). Lanes 10 and 13: PCR performed on DNA extracted from the brainstem (lane 10, VMH-GFP-mouse 1; lane 13, VMH-GFP-mouse 2). Lane 14: PCR of DNA extracted from the hypothalamus of a *Thrb*$^{flox/flox}$ mouse injected with rAAV-Cre. Lane 15: PCR of DNA extracted from the hypothalamus of an un-injected *Thrb*$^{flox/flox}$ mouse. Lane 16: PCR of DNA extracted from the hypothalamus of a wildtype mouse. Lane 17: negative control (autoclaved glass distilled water). Lane 18: HyperLadder I™ (DNA molecular weight marker).

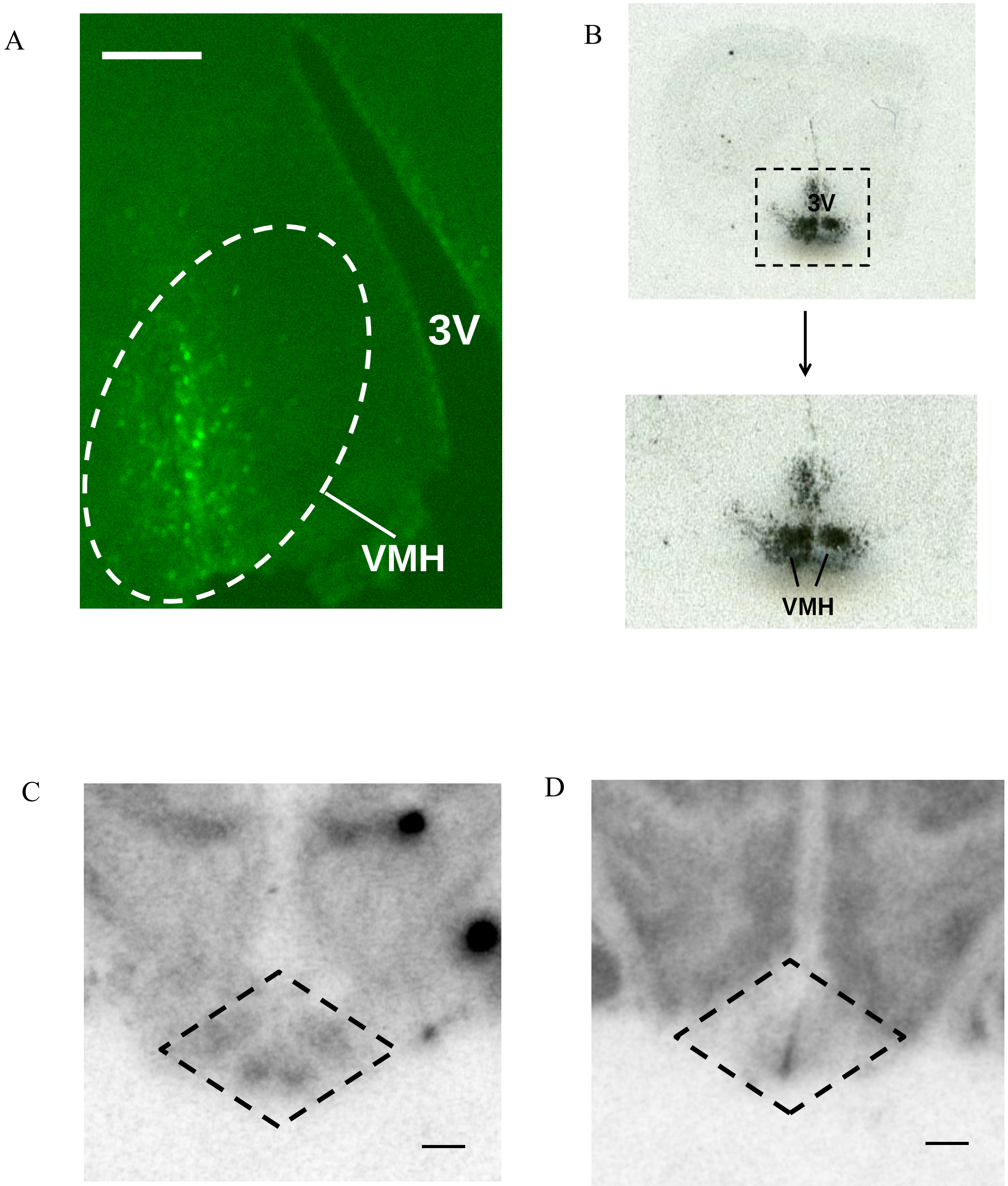

Figure S2 Localization studies, verification of rAAV-Cre transgene expression and excision of the TRβ^{flox} allele within the ventromedial hypothalamus related to figure 1

(A) GFP fluorescence within the VMH which sits adjacent to the third ventricle (3V) (scale bar represents 6μm). **(B)** Representative *in situ* hybridization image of a VMH-TRβ$^-$ mouse brain radio-labelled with woodchuck hepatitis post-regulatory element (WPRE) antisense riboprobe which localizes transgene expression to the VMH. The WPRE sequence is part of the expression cassette of rAAV vectors but is not endogenously expressed by mammalian cells. Its detection therefore confirms successful rAAV neuronal infection and transgene expression. Scale bar is 0.2mm **(C)** Representative *in situ* hybridization image of a VMH-GFP mouse brain radio-labelled with TRβ antisense riboprobe showing expression of TRβ within the VMH (hashed area). This is in comparison to the *in situ* hybridization image **(D)** showing a VMH-TRβ$^-$ mouse brain radio-labelled with *Thrb* antisense riboprobe. The lack of riboprobe binding in the VMH-TRβ$^-$ mouse brain (D) (area marked by hashed lines) in comparison to the control brain (C) suggests reduced expression of TRβ in the VMH of VMH-TRβ$^-$ mice. Scale bar 25μm.

A

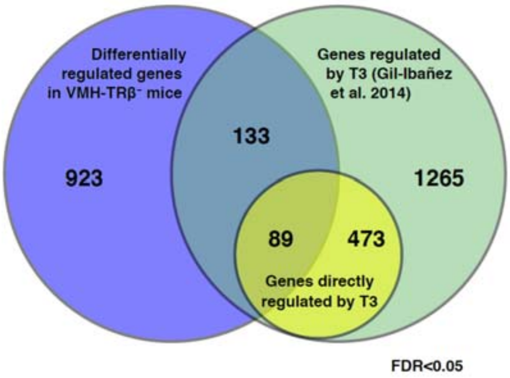

B

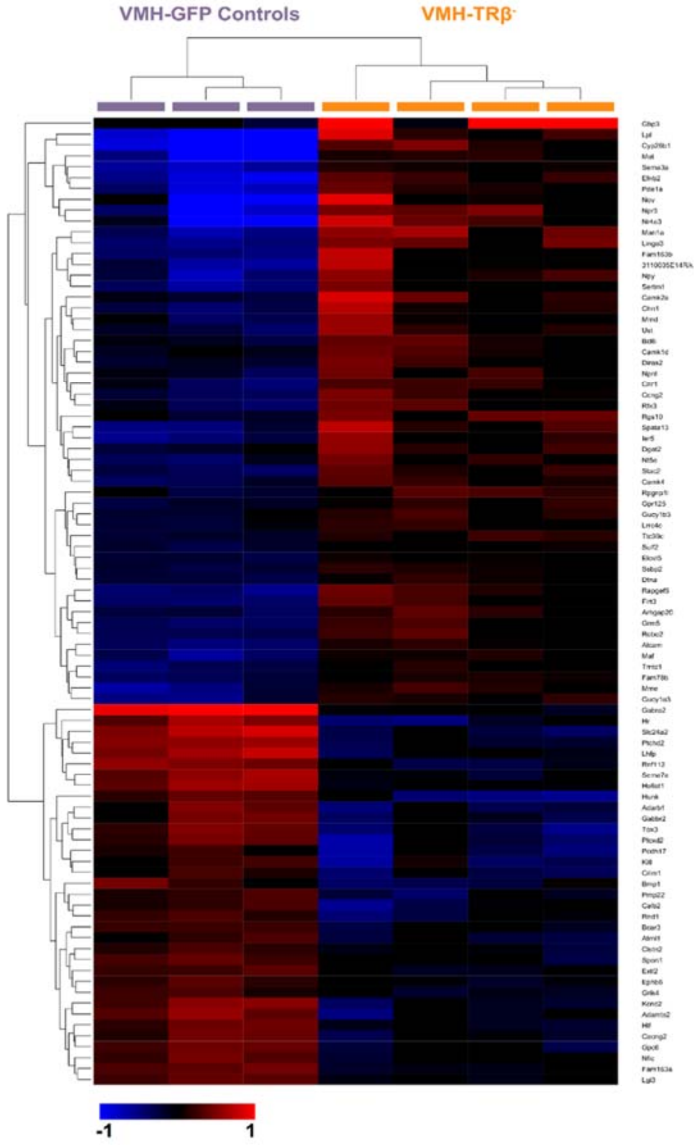

Figure S3: Venn diagram of comparison of differentially regulated gens in VMH-TRβ$^-$ mice and those reported to be T3 sensitive in cerebrocortical cells and heat map of significantly altered genes directly regulated by T3 related to figure 2

RNA was extracted from hypothalami from VMH-GFP (n=3) and VMH-TRβ$^-$ (n=4) mice and subject to RNA seq analysis.

A. Significant differential expression genes were compared to those reported by Gil-Ibaňez al 2017.

B. Significant differential expression genes were compared to those reported by Gil-Ibaňez al 2017 and shown are the 93 genes reported to be directly regulated by T3 that were significantly altered in our samples.

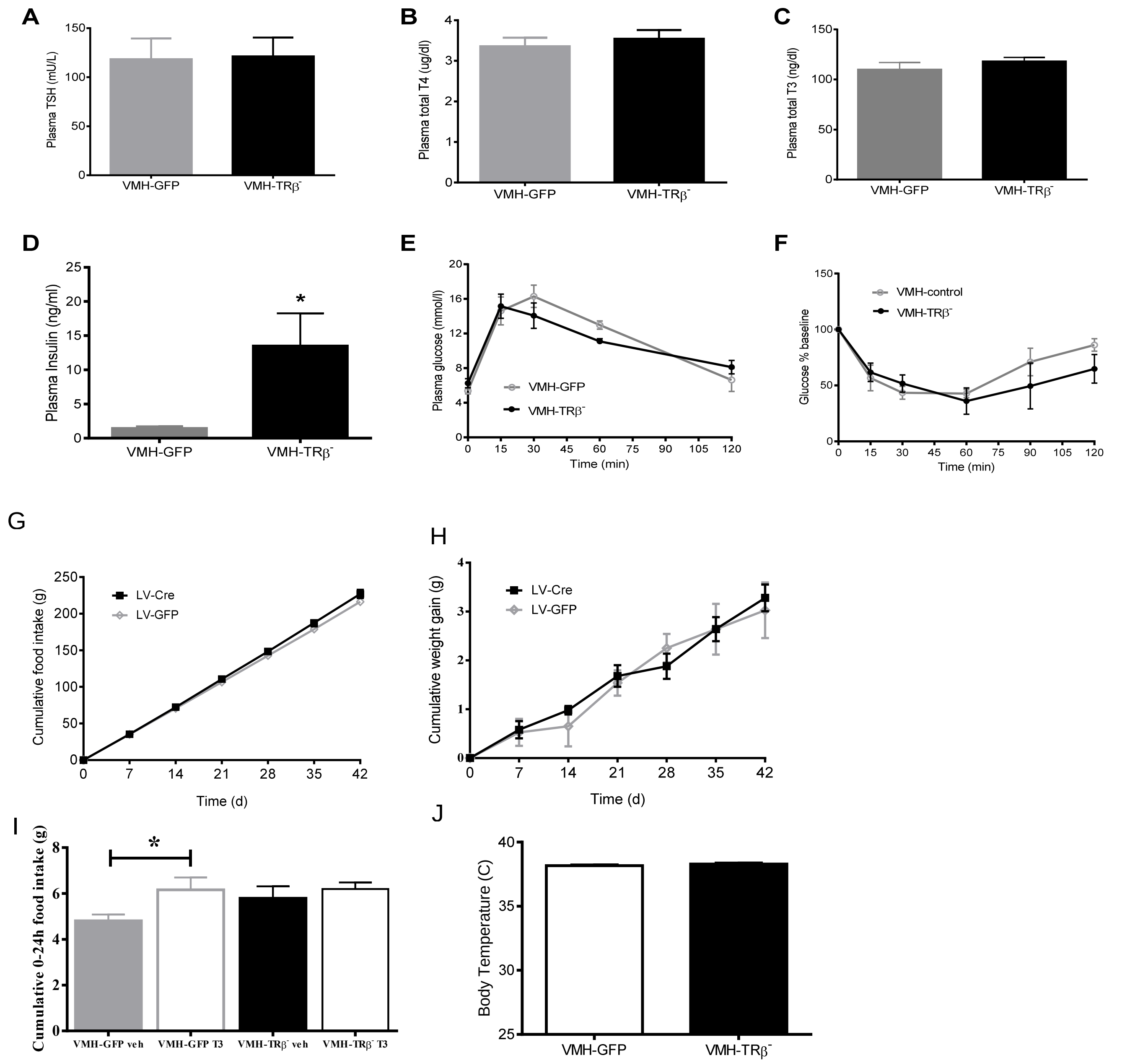

Figure S4: Systemic thyroid function and glucose homeostasis body temperature and response to exogenous T3 injection of VMH-TRβ$^-$ and VMH-GFP and effect of lateral ventricle injection of rAAV-Cre (LV-Cre) or rAAV-GFP (LV-GFP) into the lateral ventricles (LV) of *Thrb*$^{flox/flox}$ mice mice related to figure 2

(A) Plasma mTSH.
(B) Plasma total T4.
(C) Plasma total T3.
(D) Fasting plasma insulin.
(E) Glucose tolerance test performed before development of obesity in mice.
(F) Insulin tolerance test performed before development of obesity in mice.
(G) Weight change in LV-Cre and LV-GFP mice
(H) Cumulative food intake in LV-Cre and LV-GFP mice
(I) Twenty-four hour food intake in VMH-GFP or VMH-TRβ$^-$ mice in response to exogenous T3 or vehicle.
(J) Body temperature in VMH-TRβ$^-$ and VMH-GFP mice.
Results (A-C) are mean ± s.e.m. (n=7 per group). Data were analyzed by Mann-Whitney U test.
Results D are mean ± s.e.m. (n=10 per group). Results E-F are mean ± s.e.m. (n=9 per group).
Results G and H are mean ± s.e.m. (n=8-10 per group). Data were analyzed using generalized estimating equations with exchangeable correlation matrix and robust standard errors
Results I are mean ± s.e.m (n=5-7 per group) Results J are mean ± s.e.m (n=4 per group) Data were analyzed by t-test.
.

	Dark phase		Light phase	
	**VMH-GFP**	**VMH-TRβ^{-}**	**VMH-GFP**	**VMH-TRβ^{-}**
**Feeding**	19 (16-23)	21 (9-25)	14 (13-17)	9 (9-14)
**Drinking**	2 (0-3)	0 (0-0)	0 (0-1)	0 (0-0)
**Grooming**	24 (22-26)	17 (11-24)	15(10-19)	13 (4-16)
**Burrowing**	1 (0-2)	1 (0-3)	3 (0-4)	6 (1-8)
**Rearing**	2 (0-3)	0 (0-1)	0 (0-0)	0 (0-0)
**Locomotion**	21 (17-22)	**11 (7-13)****	9 (7-10)	6 (3-8)
**Sleep**	24 (19-24)	36 (32-41)	60 (42-65)	60 (48-68)
**Head down/still**	17 (11-21)	21 (13-32)	10 (3-15)	15 (13-18)

Table S3 Effect of TRβ inactivation in the VMH on mouse behavior related to figure 4. At least twenty eight days after rAAV injection, behavioral patterns were monitored continuously for sixty minutes at 08.30h, 12.30h, 16.30h, 19.30h, 00.00h and 04.00h by observers blinded to the experimental treatment. At every time point, each animal was observed for three five second periods every five minutes and the behavior noted. There was a significant reduction in nocturnal locomotor activity in VMH-TRβ^{-} compared with the control group. There was no difference in abnormal behaviors (defined by a significant increase in head down, burrowing or rearing) between VMH-TRβ^{-} and control mice. Results are median (interquartile range) (n=7-10 per group); ** P<0.01 versus control data were analyzed by Kruskal-Wallis one way analysis of variance.

Supplemental Experimental Procedures

Stereotaxic surgery

Stereotaxic surgery was performed on eight week old male *Thrb*$^{flox/flox}$ mice (Gardiner et al., 2005). The VMH injection coordinates were 1.3mm posterior, 0.4mm lateral and 6mm ventral. The LV coordinates were 0.5mm posterior, 1.1mm lateral and 2.4mm ventral. Each mouse received a 0.5 µl bilateral injection of either rAAV-Cre 7.63x10^{13} gp/ml or rAAV-GFP, 8.57x10^{13} gp/ml. Mice were individually housed at 21-23°C with a 12-h light/dark cycle with *ad libitum* access to food (RM1 diet; DS, Witham, UK) and water unless otherwise specified.

RNA seq analysis

RNA-Seq analysis was performed using hypothalamic RNA from VMH-GFP (n=3) and VMH-TRβ- (n=4) mice using Next Generation Sequencing (NGS) technologies (Imperial BRC Genomics Laboratory, Imperial College London). TruSeq Stranded mRNA libraries were multiplexed and sequenced with the average of 40 million DNA fragments per sample (100 bp paired-end reads). Quality control was performed using FastQC software (version 0.11.2). Sequencing reads were aligned to GRCm38 reference mouse genome by Tophat (version 2.0.10) using the set of known genes provided by Ensembl database (release 75) with the average alignment rate of 85%. The raw number of read pairs mapped to each Ensemble gene was calculated with HTSeq (version 0.6.0) in 'union' mode. Reads (or read pairs) that overlap more than one gene or mapped to multiple locations were discarded. Differential expression analysis was performed using EdgeR and an FDR cutoff of 0.05 was used to generate the lists of DE genes. The lists of T3 responsive genes and direct T3 responsive genes were obtained from Gil-Ibañez et al. 2017 and overlapped with DE expressed genes from the present study. Ingenuity Pathway Analysis was performed using the resultant sets of DE genes. A heatmap comparing gene expression in 89 direct T3 responsive genes in the present study was generated using GeneSpring.

Quantitation and distribution of white adipose tissue by MRI

VMH-TRβ$^-$ and VMH-GFP mice (n=3 per group) were scanned using a 4.7 Tesla Varian INOVA imaging system. SliceOmatic software (version 4.2) was used to separate and quantify tissue volumes (Mystkowski et al., 2000). Quantitation of fat depots was normalized to total body fat and total body fat was normalized to body weight.

Glucose and insulin tolerance tests

Glucose and insulin tolerance tests were carried out as previously described (Bewick et al., 2009). Plasma glucose was measured using the Acensia Contour blood glucose monitoring system (Bayer HealthCare, Newbury, U.K.).

Peripheral (subcutaneous) administration of T3 (75nmol/kg) and food intake

In a randomized crossover, VMH-TRβ$^-$ mice and VMH-GFP mice control mice (n=5-7 per group) received either subcutaneous T3 (75nmol/kg) or vehicle as previously described (Kong et al., 2004) and food intake measured.

GFP visualization

Animals were terminally anaesthetized and the brains dissected, incubated and sliced as previously described (Gardiner et al., 2005). Fluorescence was detected by a Zeiss deconvoluting microscope (Axiovert S100 TV, Carl Zeiss, Jena, Germany) using a FITC filter. Images were acquired using a MetaMorph imaging system (Universal Imaging, West Chester, USA) as previously described (Gardiner et al., 2005).

Pair-feeding of VMH-TRβ$^-$ mice to the food intake of VMH-GFP mice

Twenty eight days after rAAV injection, VMH-TRβ$^-$ mice were pair-fed to the mean daily food intake of a weight matched VMH-GFP litter mate (n=9 per group). After 5 weeks of pair-feeding, *ad libitum* feeding was re-instated for a further 4 weeks.

Plasma assays

Total T4, T3 and TSH were measured by radioimmunoassay (RIA) (Pohlenz et al., 1999). Fasting leptin and fasting insulin were measured by enzyme linked immunosorbent assay (Crystal Chem, IL).

Quantitation of *Ucp1* mRNA expression in BAT by northern blot analysis

RNA was extracted from inter-scapular BAT of VMH-TRβ$^-$ and VMH-GFP mice (n=7-11 per group) and *Ucp1* mRNA expression determined by northern blot analysis as previously described (Smith et al., 2008).

Measurement of energy expenditure

The study commenced 21 days after rAAV injection. One week and 6 weeks into the study, metabolic parameters were measured for 24h (12h light phase, 12h dark phase) by indirect calorimetry using an open-circuit Oxymax system of the Comprehensive Lab Animal Monitoring System from Columbus Instruments (Columbus, OH, USA). Animals (n=5-6 per group) were maintained at 21-23°C with a 12-h light/dark cycle with *ad libitum* access to food (RM1 diet; DS, Witham, UK) and water. To measure oxygen consumption and carbon dioxide production exhaust air from each tight chamber was sampled for 1min at 30min intervals. Oxygen consumption and carbon dioxide production were normalized to surface area (Tschop et al., 2011) (body weight to the power of 0.75). The ambulatory activity of each animal was assessed simultaneously using the optical beam technique as previously described (Gardiner et al., 2010).

Behavioral Analysis

At least 28 days after rAAV injection, behavioral patterns of VMH-TRβ^- and VMH-GFP mice (n=7-10 per group) were monitored continuously for sixty minutes at 08.30h, 12.30h, 16.30h, 19.30h, 00.00h and 04.00h, by observers blinded to the experimental treatment. At every time point, each animal was observed for three, five second periods every five minutes and the behavior noted. Behavior was classified into eight categories: feeding, drinking, grooming, burrowing, rearing, locomotion, sleep, head down/still as previously described (Fray et al., 1980; Abbott et al., 2001). Abnormal behavior was defined by a significant increase in head down, burrowing or rearing as previously described (Abbott et al., 2001).

Supplemental References

Abbott, C.R., Rossi, M., Wren, A.M., Murphy, K.G., Kennedy, A.R., Stanley, S.A., Zollner, A.N., Morgan, D.G., Morgan, I., Ghatei, M.A., *et al.* (2001). Evidence of an orexigenic role for cocaine- and amphetamine-regulated transcript after administration into discrete hypothalamic nuclei. Endocrinology *142*, 3457-3463.

Bewick, G.A., Kent, A., Campbell, D., Patterson, M., Ghatei, M.A., Bloom, S.R., and Gardiner, J.V. (2009). Mice with hyperghrelinemia are hyperphagic and glucose intolerant and have reduced leptin sensitivity. Diabetes *58*, 840-846.

Fray, P.J., Sahakian, B.J., Robbins, T.W., Koob, G.F., and Iversen, S.D. (1980). An observational method for quantifying the behavioural effects of dopamine agonists: contrasting effects of d-amphetamine and apomorphine. Psychopharmacology (Berl) *69*, 253-259.

Mystkowski, P., Shankland, E., Schreyer, S.A., LeBoeuf, R.C., Schwartz, R.S., Cummings, D.E., Kushmerick, M,, Schwartz, M.W. (2000). Validation of whole-body magnetic resonance spectroscopy as a tool to assess murine body composition. Int J Obes Relat Metab Disord 24, 719-24.

Pohlenz, J., Maqueem, A., Cua, K., Weiss, R.E., Van Sande, J., and Refetoff, S. (1999). Improved radioimmunoassay for measurement of mouse thyrotropin in serum: strain differences in thyrotropin concentration and thyrotroph sensitivity to thyroid hormone. Thyroid 9, 1265-1271.

Tschöp, M.H., Speakman, J.R., Arch, J.R., Auwerx, J., Brüning, J.C., Chan, L., Eckel, R.H., Farese, R.V. Jr, Galgani, J.E., Hambly, C., et al. (2011). A guide to analysis of mouse energy metabolism. Nat Methods. 9, 57-63.
